# A novel tool for assessing pediatric emergency care in low- and middle-income countries: a pilot study

**DOI:** 10.1186/s12245-024-00802-2

**Published:** 2025-01-16

**Authors:** Sonia Y. Jarrett, Andrew Redfern, Joyce Li, Camilo E. Gutierrez, Priyanka Patel, Olurotimi Akinola, Michelle L. Niescierenko

**Affiliations:** 1 Division of Emergency Medicine, Boston Children’s Hospital, 300 Longwood Ave, Boston, MA 02115 USA; 2 Department of Paediatrics & Child Health, Stellenbosch University and Tygerberg Hospital, Cape Town, South Africa; 3 Emergency Medicine Division, Children’s National Hospital, 111 Michigan Ave NW, Washington, DC 20010 USA; 4 Coast General Teaching and Referral Hospital, Mombasa, Kenya; 5 Department of Emergency Medicine, University College Hospital, Queen Elizabeth Road, Agodi, Ibadan 200285 Nigeria

**Keywords:** Pediatric emergency medicine, Pediatric readiness, Low-income countries, Middle-income countries, Health resources / supply and distribution

## Abstract

**Background:**

Globally, most children seek emergency care at general rather than specialized pediatric emergency departments. There remains significant variation in the provision of pediatric emergency care, particularly in resource-constrained settings. The objective of this study is to pilot a self-assessment tool to evaluate pediatric emergency care capabilities in low- and middle-income country (LMIC) hospitals on the African Continent.

**Methods:**

This was a prospective cross-sectional descriptive study using a convenience sample of sub-Saharan African hospitals. The assessment tool was developed by operationalizing the technical contents of existing standards and guidelines from international bodies including the World Health Organization and International Federation of Emergency Medicine. The pilot was conducted at emergency departments located across different regions on the African continent. Descriptive statistics were used to evaluate different domains of pediatric emergency care capabilities including pediatric triage, protocols, staffing, training, equipment, consumables, and medicines.

**Results:**

Sixteen hospitals with emergency departments completed the assessment tool (participation rate of 76%). The hospitals were in nine different countries across four regions of sub-Saharan Africa. National/academic hospitals comprised 56.3% of the participating hospitals. The majority, 44%, of these hospitals saw pediatric patient volumes of 2,000–4,999 patients per year. Dedicated pediatric triage spaces and resuscitation spaces were available at 37.5% and 56.3%, respectively. Formal pediatric resuscitation guidelines were used at 62.5%. Doctors on the self-assessment teams came from primarily pediatrics and general practitioner training backgrounds (both 68.8%). Basic respiratory and airway support equipment (e.g. oxygen, bag-valve mask devices) were available in all participating hospitals, whereas advanced airway equipment (e.g. pediatric intubation equipment) was available in 37.5% of hospitals. Most medicines from the World Health Organization Essential Medicines list were available at participating hospitals.

**Conclusions:**

To date, this is the first assessment tool dedicated to the comprehensive evaluation of pediatric emergency care in LMICs. This pilot provides a first approach to evaluate pediatric emergency healthcare capabilities in the hospital setting with future directions to improve the tool based on qualitative feedback.

**Supplementary Information:**

The online version contains supplementary material available at 10.1186/s12245-024-00802-2.

## Background

Emergency care for children presents challenges to clinicians and hospitals that primarily treat adults. In the United States (US), 83% of children who seek emergency care present to general emergency departments (EDs) rather than specialized pediatric EDs [1]. In low- and middle-income countries (LMICs), EDs rarely serve pediatric patients exclusively [2]. Due to the unique anatomic, physiologic and developmental needs of children, many hospitals are not prepared to treat pediatric patients [3–5].

To address gaps in providing effective pediatric emergency care, in 2013 the National Pediatric Readiness Project (NPRP) launched an initiative in the US to evaluate hospitals’ “pediatric readiness,” or preparedness to care for pediatric patients [6]. The NPRP uses an assessment to score hospitals’ pediatric readiness [7]. Critically ill children presenting to hospitals with a high pediatric readiness score had decreased in-hospital mortality [8]. The association of increased pediatric readiness and improved clinical outcomes has significant implications for children globally.

Globally, there remains variation in the provision of pediatric emergency medicine services [9]. To promote best practices, the International Federation of Emergency Medicine (IFEM) Pediatric Emergency Medicine Special Interest Group (PEMSIG) developed the expert consensus document “Standards of Care for Children in Emergency Departments” for both low- and high-income countries [10, 11]. To improve early recognition of critically ill children and provide stabilizing treatment, the World Health Organization (WHO) developed the Emergency Triage Assessment and Treatment (ETAT) guidelines in 2005 [12]. Implementation of ETAT has reduced in-hospital pediatric deaths in multiple LMIC contexts [13–15]. These standards, however, do not have associated tools for EDs to evaluate their own pediatric emergency care.

Few studies examine pediatric emergency care outside of high-income countries in North America and Europe. In 2013, the African Federation of Emergency Medicine (AFEM) developed an Emergency Care Assessment Tool (ECAT) to evaluate how EDs treated patients of all ages in four LMICs: Cameroon, Uganda, Egypt, and Botswana [16]. The ECAT did not collect nuanced details about pediatric emergency care [16]. Studies in Singapore, Lebanon, Latvia, and Saudi Arabia demonstrated limited generalizability of using the NPRP assessment to evaluate pediatric readiness outside the US [17–20]. In Latin America, EDs have wide variability in resources and care processes despite having mostly pediatric-trained clinicians evaluating children [21].

To our knowledge, there is no assessment tool dedicated to the evaluation of pediatric emergency care in resource-constrained settings. Pediatric emergency healthcare capabilities (PEHC) refer to a hospital’s ability to triage, treat, and stabilize a pediatric patient for the first 24 h after presentation, or prior to disposition. These stages of care focus on the early recognition and stabilization of acutely ill children to prevent deaths which often occur within 24 h of presentation [22].

The objective of this study is to develop and pilot a new online self-assessment tool to evaluate PEHC in LMIC hospitals on the African continent. The goal of the assessment tool is to serve as a framework for LMICs to improve pediatric emergency care at the hospital or health system level.

## Methods

### Assessment tool development

A prospective cross-sectional descriptive study was performed to pilot a novel PEHC Self-Assessment Tool (PEHC-SAT). The PEHC-SAT operationalized technical contents of existing standards and guidelines into a quantifiable and measurable assessment of the components of pediatric emergency care. A literature search conducted in January and June of 2021 screened for pre-existing pediatric emergency care guidelines. Search terms included “readiness,” “preparedness,” “pediatric,” “emergency,” “resource-limited,” or “low resource.” The search yielded 753 articles, 61 addressed pediatric emergency medicine. Guidelines and standards from these articles by internationally recognized bodies were selected, including the NPRP Assessment [7]; IFEM “Standards of Care for Children in Emergency Departments” [10]; WHO ETAT [12]; the Royal College of Pediatrics and Child Health (RCPCH) “Facing the Future: Standards for Children in Emergency Care Settings” [23]; WHO Essential Medicines for Children 2019 [24].

For tool development, the project team included subject matter experts consisting of an IFEM leader, a US Pediatric Readiness Special Interest Group task force leader, and physicians with backgrounds in pediatric emergency medicine, pediatrics, and emergency medicine from hospitals in Nigeria, Kenya, and South Africa. The WHO guidelines and IFEM standards were used prominently because they were designed for LMIC settings [19, 23, 25]. The PEHC-SAT also incorporated methodology from the NPRP Assessment [7] and WHO Harmonized Health Facility Assessment [26].

The PEHC-SAT evaluated the stages of triage, treatment, and stabilization within a patient’s first 24 h of presentation. These stages of emergency care were applied to patients arriving at a hospital seeking care from the emergency department without an appointment.

**Fig. 1:**
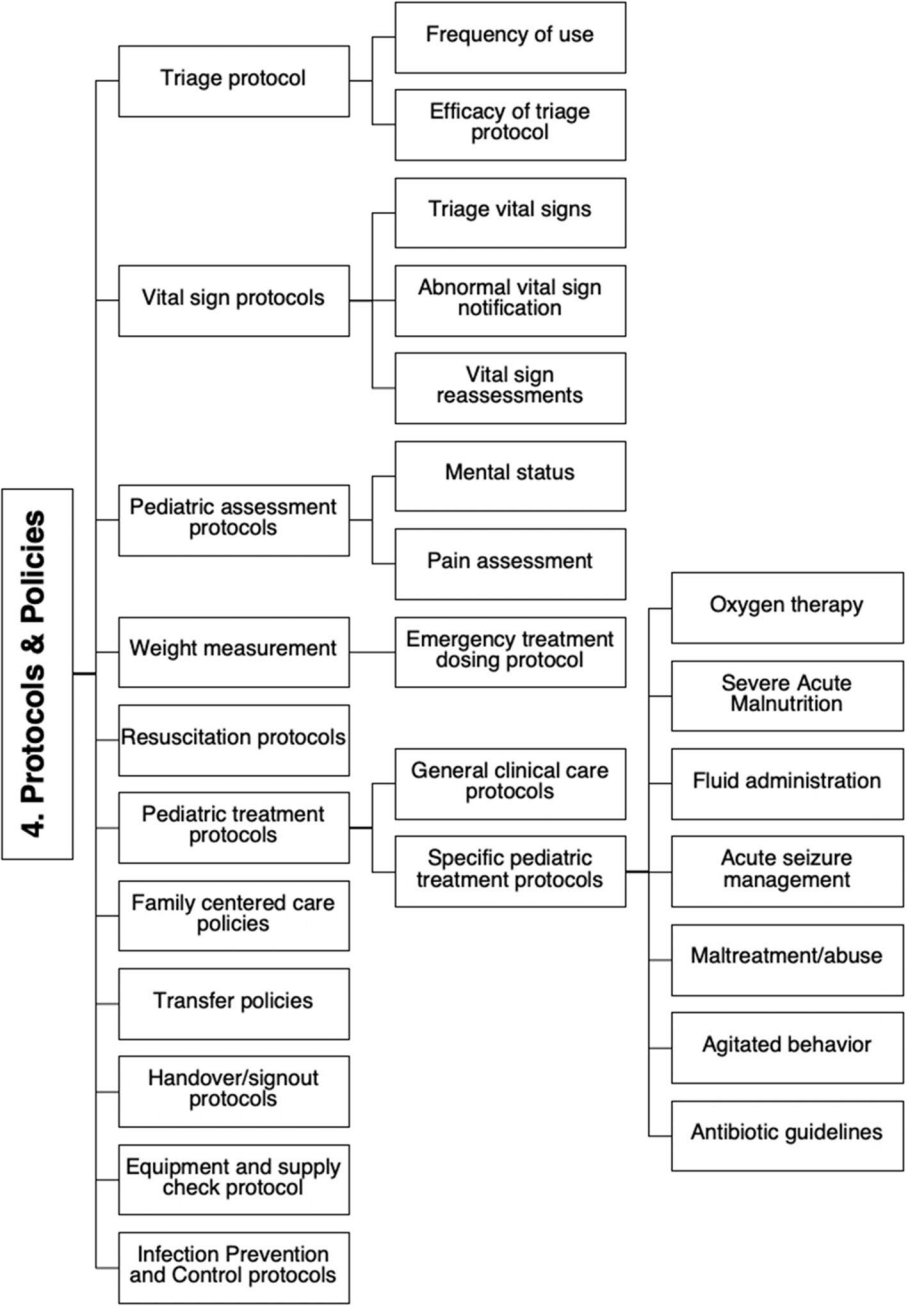
Example outline of the “protocols and policies” assessment tool domain

The PEHC-SAT collected basic participant demographic information and general characteristics about the participating hospitals. The PEHC-SAT assessed five domains: infrastructure, staffing and training, policies and protocols, equipment and consumables, and medicines. Each domain was further divided into subdomains (Fig. 1). Domain overviews are available in Appendix 1.

The tool underwent iterative review and revision based on subject matter experts’ feedback. The final version was written in English and consisted of 379 questions total (360 closed-response) with branching logic embedded throughout. To minimize barriers to completion, only demographic questions were mandatory.

### Data collection

The open-source software KoboToolbox [27] served as the PEHC-SAT platform. KoboToolbox is the United Nations’ endorsed international standard for online data collection [27]. Once participants accessed the tool through a link and loaded it on a browser, they could complete the assessment online or offline, which was advantageous for settings with limited internet access. An accompanying Standard Operating Procedure (SOP) instructed users on how to complete the assessment (Appendix 2).

### Study participants and recruitment

This study assessed hospitals with EDs or designated areas for emergency care for pediatric patients. Pediatric patients were defined as newborn up to 18 years of age. The study excluded outpatient clinics, inpatient care areas or intensive care units. The self-assessment tool was not intended to be used for evaluation of the ability to provide patient care after the initial 24 h of treatment and stabilization.

Hospitals were recruited across the African continent using the Boston Children’s Hospital Global Health Program (BCHGHP) network of partnerships and project subject matter expert contacts. Outreach to a convenience sample of 32 hospitals included up to three recruitment emails with a description of the project and an accompanying flyer. Interested individuals at these hospitals attended a one-hour Zoom video-conference informational meeting to introduce the PEHC-SAT.

The PEHC-SAT was completed by an individual or group of staff from participating hospitals, defined as a single healthcare worker (HCW), a group of HCWs, and/or management identified by the participating hospital that could provide the requested information. The informational video conference and SOP provided guidance on the number of participants required. All participants identified their qualifications (e.g. doctor, nurse) in the PEHC-SAT.

The project was reviewed and approved for exemption by the institutional review board at Boston Children’s Hospital. The assessment tool did not collect any patient data or identifiable information about HCWs. The data containing hospital demographic information was available only to the study team that conducted all the analyses. Participation was voluntary and participants reviewed an agreement prior to initiating the assessment. Based on their responses, hospitals received an individualized report summarizing their own ED’s PEHC with associated relevant informational resources.

### Assessment-tool deployment

After the informational meeting, participants received the PEHC-SAT KoboToolbox link. The goal was to complete the assessment within one month. To improve completion rates, participants received up to 3 weekly reminder emails.

### Outcome measures

The primary outcome was evaluation of PEHC of participating hospitals based on quantitative data obtained from the completion of the self-assessment tool. As a pilot study, validation of the PEHC-SAT was outside the scope of this study.

### Statistical analysis

Data was exported from KoboToolbox to Excel for analysis. Data were analyzed from May to August 2022 after all participating EDs completed the PEHC-SAT. Descriptive statistics were used to characterize PEHC across hospitals and within subgroups of hospitals based on region, hospital type, and patient volume. Statistical methods included frequencies and percentages for categorical data and medians for quantitative variables within each domain. Blank responses were reviewed to evaluate the extent of assessment tool completion. Blank responses were considered missing information with the rationale that the information is unknown either because it is not available or easily accessible.

## Results

### Participants

After email outreach to 32 potential participating hospitals, 21 hospitals indicated interest in the pilot (66% response rate) with 11 non-responders. Sixteen out of 21 hospitals completed the assessment tool (76% completion rate). Barriers to completion of the assessment tool included inability to obtain hospital-specific approval (*N* = 3), technological issues (*N* = 1), and ED renovations (*N* = 1) that necessitated postponement of the pilot.

Across the 16 participating sites, 36 individuals in multi-disciplinary teams provided responses to the assessment tool. The professional titles of participants included doctor (72%, 26/36), nurse (11.1%, 4/36), pharmacist (8%, 3/36), physician assistant (2.8%, 1/36), administrator (2.8%, 1/36), and physical therapist (2.8%, 1/36). Participant group sizes ranged from 1 to 10 individuals (one person *N* = 10, two people *N* = 2, three people or more *N* = 4).

The duration of time from starting the assessment to submission ranged from 0.6 to 411.5 h (including time without active data entry), with a median of 25.9 h (interquartile range: 2.6–174.7 h). The median time for tool completion for individuals was 25.9 h (range 0.6 to 411.5 h) and 82.2 h for groups (range 1.2–219.5 h).

Of blank responses, 23% (32/140) were not branching logic questions. The number of blank responses were distributed across domains: demographics *N* = 15, hospital characteristics *N* = 2, protocols and policies *N* = 1, staffing and training *N* = 26, equipment and consumables *N* = 7, and medicines *N* = 19.

### Hospital characteristics

Of the participating hospitals, 56.3% were national or academic hospitals and 31.3% were district hospitals. 75% of hospitals were public or government-run. The greatest regional representation was Western (56.3%) and Southern Africa (25%) (Table 1). The upper age limit for pediatric patients seen at the participating hospitals ranged from 12 to 18 years old (median 14 years old).

**Table 1:** Hospital characteristics, infrastructure, and services

Hospital Regional Location	% of Responding EDs, (*n*/*N*^a^)
Eastern Africa	12.5 (2/16)
Western Africa	56.3 (9/16)
Central Africa	6.3 (1/16)
Southern Africa	25 (4/16)
Hospital Classification	
District hospital	31.3 (5/16)
Provincial or regional hospital (secondary referral hospital)	12.5 (2/16)
National or academic hospital (tertiary referral hospital)	56.3 (9/16)
Hospital Managing Authority	
Public/government	75 (12/16)
Private for profit	6.3 (1/16)
Non-governmental organization/not-for-profit	6.3 (1/16)
Mission/faith-based	12.5 (2/16)
ED Pediatric Patient Volume (Per Year)	
< 2000 (low volume)	12.5 (2/16)
2,000–4,999 (medium volume)	43.8 (7/16)
5,000–9,999 (medium high volume)	25 (4/16)
> 10,000 (high volume)	18.8 (3/16)
ED Electricity Source	
National or local electric grid	100 (16/16)
Hospital generator	75 (12/16)
Solar energy	12.5 (2/16)
Reliable backup power source	81.3 (13/16)
ED Medical Records	
Paper charts	87.5 (14/16)
Electronic health records	31.3 (5/16)
Child health cards	25 (4/16)
ED Water Supply & Plumbing	
Piped from government/municipal supply	68.8 (11/16)
Bore hole	25 (4/16)
Well	6.3 (1/16)
Running water available in ED	87.5 (14/16)
Pediatric Patient Care Spaces	
Dedicated triage space for pediatric patients	37.5 (6/16)
Dedicated resuscitation space for unstable pediatric patients	56.3 (9/16)
Pediatric ED Patient Services & Capabilities	
24 h pharmacy services available for pediatric patients	62.5 (10/16)
Pharmacy Characteristics	
Shared ED and hospital pharmacy	43.8 (7/16)
Dedicated ED pharmacy	31.2 (5/16)
Medication cabinet/store room in the ED	25 (4/16)
24 h lab services available to pediatric patients	87.5 (14/16)
Blood typing	93.8 (15/16)
Complete blood count	93.8 (15/16)
Blood chemistry	93.8 (15/16)
Microbiology staining	81.3 (13/16)
Microbiology cultures	68.8 (11/16)
Blood gas measurement	62.5 (10/16)
Intubation and management of ventilated pediatric patients	75 (12/16)
Radiology Services for Pediatric Emergency Patients	
X-ray	81.3 (13/16)
X-ray available for pediatric patients 24 h per day	76.9 (10/13)
Ultrasonography (US)	75 (12/16)
US available 24 h	41.7 (5/12)
Computed Tomograhy (CT) Scanner	56.3 (9/16)
CT available 24 h	44.4 (4/9)
Magnetic Resonance Imaging (MRI)	37.5 (6/16)
MRI available 24 h	50 (3/6)

^a^Denominator reflects the number of participating hospitals that answered the question

### Infrastructure and services

Of the participating hospitals, 87.5% (14/16) had inpatient pediatric wards, 62.5% (10/16) had nurseries, 68.8% (11/16) had neonatal intensive care units, and 31.3% (5/16) had pediatric intensive care units. One participating hospital had no inpatient pediatric services.

Three EDs (18.8%, 3/16) had access to current online medical references. Medical records in 87.5% (14/16) of the EDs were paper charts. Pharmacy services for pediatric ED patients included medications dispensed from a shared pharmacy for the ED and hospital (43.8%, 7/16). Ten hospitals (62.5%) had pharmacy services available 24 h a day for pediatric ED patients.

Twenty-four-hour laboratory services were available at 87.5% (14/16) of hospitals. Blood transfusion was available in all EDs, though the ability to perform transfusions within 2 h more than 75% of the time was available at 62.5% (10/16) of EDs. The most available radiology services for pediatric patients were X-ray (81.3%) and ultrasonography (75%) (Table 1).

### Protocols and policies

Of the 68.8% of EDs with a formal pediatric triage protocol, the South African Triage Score (54.5%, 6/11) and WHO ETAT guidelines (36.4%, 4/11) were most used. When no formal pediatric triage process was in place, 80% (4/5) of those EDs had a list of emergency signs for pediatric patients who require immediate treatment. Ten EDs (62.5%) used a formal pediatric resuscitation protocol, 40% used Pediatric Advanced Life Support (PALS), 20% used Advanced Pediatric Life Support (APLS), 10% used the WHO ETAT.

Protocols that were least commonly available included pain assessment (31.3%, 5/16), protocols for acute mental health complaints (18.8%, 3/16), and suicide screening (6.3%, 1/16) (Table 2).

**Table 2:** Comparison of Pediatric Emergency protocols and policies based on patient volume

	All Categories		Low pediatric ED volume (< 2,000)^a^		Medium volume (2,000–4,999)^a^		Medium-high volume (5,000–9,999)^a^		High volume (≥ 10,000)^a^	
	*N* = 16		*N* = 2		*N* = 7		*N* = 4		*N* = 3	
Pediatric Policy	No.	%	No.	%	No.	%	No.	%	No.	%
Pediatric triage protocol	11	68.8	2	100	4	57.1	3	75	2	66.7
Notification of abnormal vital signs	15	93.8	2	100	6	85.7	4	100	3	100
Vital sign reassessments	12	75	1	50	5	71.4	4	100	2	66.7
Pain assessment	5	31.3	0	0	3	42.9	1	25	1	33.3
Weight in kilograms	15	93.8	1	50	7	100	4	100	3	100
Drug dosing based on kilograms	16	100	2	100	7	100	4	100	3	100
Pre-calculated emergency drug doses based on estimated weight	6	37.5	1	50	3	42.9	1	25	1	33.3
Pediatric resuscitation guideline	10	62.5	1	50	3	42.9	4	100	2	66.7
Pediatric clinical care protocols	11	68.8	2	100	4	57.1	3	75	2	66.7
Infection control protocols for patient isolation	9	56.3	1	50	3	42.9	3	75	2	66.7
Antimicrobial resistance (AMR) program	7	43.8	0	0	2	28.6	3	75	2	66.7
Family centered care: parent presence during all aspects of emergency care, including resuscitation	6	37.5	2	100	1	14.3	2	50	1	33.3
Pediatric death in the ED	11	68.8	2	100	4	57.1	4	100	1	33.3
Transfer guideline	6	37.5	2	100	2	28.6	2	50	0	0
Equipment & supply verification	10	62.5	1	50	4	57.1	3	75	2	66.7
Reduced dose radiation	8	50	1	50	4	57.1	2	50	1	33.3
Reporting suspected child maltreatment/abuse	9	56.3	2	100	3	42.9	3	75	1	33.3
Suicide screening	1	6.3	1	50	0	0	0	0	0	0
Behavioral health protocols for agitation	3	18.8	1	50	1	14.3	1	25	0	0

^a^Estimated volume of ED pediatric patients per year

### Staffing and training

Ten of the sixteen hospitals (62.5%) had a dedicated doctor staffing the ED 24 h a day. Training background for ED doctors were primarily pediatrics (68.8%, 11/16) and general/family practitioners (68.8%, 11/16) (multiple responses were available for selection). Subspecialists had varying availability for consultation (Table 3). Mental health providers were available at 50% (8/16) of EDs.

**Table 3:** Staffing and training based on patient volume

	All Categories		Low pediatric ED volume (< 2,000)^a^		Medium volume (2,000–4,999)		Medium-high volume (5,000–9,999)		High volume (≥ 10,000)	
	*N* = 16		*N* = 2		*N* = 7		*N* = 4		*N* = 3	
Staffing & Training	No.	%	No.	%	No.	%	No.	%	No.	%
Designated triage staff 24 h/day	9	56.3	0	0	2	28.6	4	100	3	100
Triage staff training	13	81.3	2	100	4	57.1	4	100	3	100
Dedicated doctor for ED 24 h/day	10	62.5	2	100	4	57.1	3	75	1	33.3
Lead doctor “pediatric champion”	8	50	1	50	5	71.4	1	25	1	33.3
Lead nurse “pediatric champion”	7	46.7	0	0	5	71.4	1	25	1	33.3
Doctor training background (certification)										
Emergency Medicine	3	18.8	0	0	2	28.6	1	25	0	0
Pediatrics	11	68.8	1	50	5	71.4	3	75	2	66.7
Pediatric Emergency Medicine	3	18.8	0	0	3	42.9	0	0	0	0
Internal Medicine	4	25	0	0	2	28.6	2	50	0	0
General/Family Practitioner	11	68.8	2	100	4	57.1	3	75	2	66.7
Available subspecialist consultation										
Surgery	13	81.3	1	50	6	85.7	3	75	3	100
Orthopedics	11	68.8	1	50	5	71.4	2	50	3	10
Cardiology	7	43.8	0	0	4	57.1	1	25	2	66.7
Neurology	7	43.8	0	0	4	57.1	1	25	2	66.7
Infectious Diseases	7	43.8	0	0	4	57.14	1	25	2	66.7
Other surgical subspecialties (Neurosurgery, Urology)	8	50	1	50	4	57.1	1	25	2	66.7
Other medical subspecialties (Endocrinology, Nephrology)	9	56.3	0	0	5	71.4	2	50	2	66.7
Staff receive training for the following skillsets^a^:										
C-spine stabilization	11	68.8	2	100	4	57.1	3	75	2	66.7
Intraosseous (IO) access: doctors	16	100	2	100	7	100	4	100	3	100
IO access: nurses	2	12.5	0	0	2	28.6	0	0	0	0
Cardiopulmonary resuscitation	16	100	2	100	7	100	4	100	3	100
Stopping active bleeding	14	87.5	2	100	5	71.4	4	100	3	100
Acute mental health needs assessment	2	12.5	1	50	0	0	1	25	0	0
Recognition of child abuse	9	56.3	2	100	4	57.1	1	25	2	66.7
Reporting child abuse based on local laws	8	50	2	100	4	57.1	2	50	0	0
Supporting caregivers/parents in response to a pediatric patient death	8	50	2	100	5	71.4	1	25	0	0

^a^Refers to both doctors and nurses unless otherwise specified

For resuscitation training, greater than 56.3% of doctors and nurses completed training in pediatric basic life support (9/16). In 50% (8/16) of EDs, a doctor or nurse with advanced pediatric life support skills (e.g. PALS, APLS) was available in the ED more than 50% of the time. Advanced life support training was available to staff at 62.5% (10/16) of EDs. Doctors or nurses were available more than 50% of the time to perform the following airway skillsets in EDs with the following frequencies: bag-valve mask ventilation 100% (16/16), surgical airway (e.g. needle cricothyrotomy) 91.7% (11/16), basic airway maneuvers (jaw thrust, chin tilt) 75% (12/16), oral airway insertion 75% (12/16), nasal airway insertion 68.8% (11/16), endotracheal intubation 56.3% (9/16).

Continuing medical education opportunities for staff were weekly in 43.8% (7/16) of EDs for doctors, 30% (5/15) for nurses, and 30% (5/15) for support staff (respiratory therapists, pharmacists).

50% of EDs (8/16) had a doctor “pediatric champion,” and 7 of those 8 EDs also had a nurse “pediatric champion” (87.5%, 7/8) who served as leaders that raise awareness of the special emergency needs of children [22].

### Equipment and consumables

**Fig. 2:**
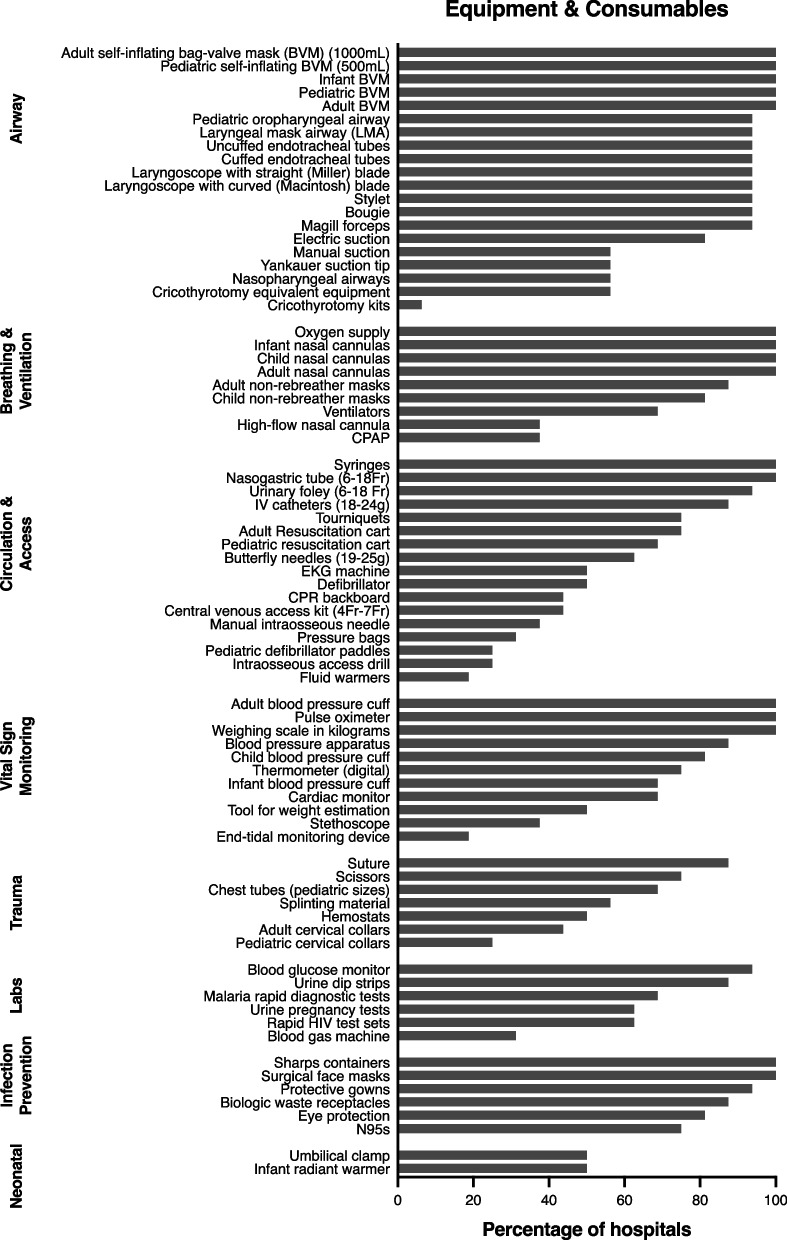
Availability of equipment and consumables by category

Basic respiratory and airway support equipment such as oxygen, pediatric bag-valve masks, and nasal cannulas were widely available (100% of EDs). Advanced respiratory support including high-flow nasal cannula and continuous positive airway pressure (CPAP) were available at 37.5% (6/16) of EDs. Resuscitation equipment availability varied, with 68.8% (11/16) of EDs with pediatric resuscitation trollies/carts, 50% (8/16) with defibrillators (25%, 4/8, with pediatric defibrillator paddles), 25% (4/16) with intraosseous drills (Fig. 2). There was no clear association between equipment availability and annual pediatric ED volumes.

### Medicines

**Fig. 3:**
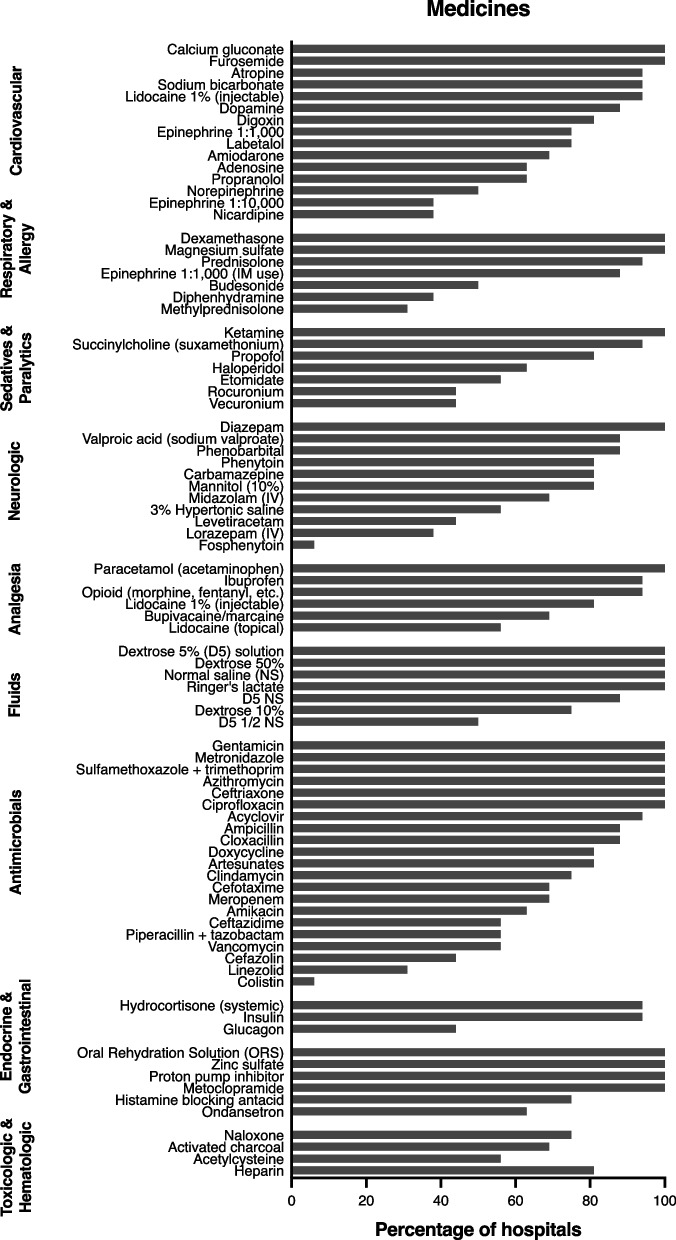
Medicine availability by category

Most medicines from the WHO Essential Medicines were available at the participating EDs except for certain paralytic agents, anti-epileptics, and antibiotics (Fig. 3).

## Discussion

The PEHC-SAT is the first known assessment tool to evaluate LMIC EDs’ pediatric capabilities on the African continent. The tool provides a new, comprehensive overview of pre-existing pediatric emergency care across five domains: infrastructure and services, protocols and policies, staffing and training, equipment and consumables, and medicines. Overall, EDs across sub-Saharan Africa, including those at tertiary referral hospitals, varied in their capabilities to evaluate and treat pediatric emergency patients.

The study included representation from four regions of sub-Saharan Africa. There was a large proportion of tertiary referral hospitals based in Western Africa, a result of convenience sampling by existing partnerships through the BCHGHP. These partnerships involve academic collaborations and workforce capacity strengthening rather than provision of equipment or staffing, potentially impacting PEHC in training or protocol domains. Most of the participating hospitals had inpatient pediatric units which may have increased overall PEHC. The tool results indicate that even tertiary hospital EDs have opportunities to adopt standardized resuscitation guidelines, promote continued medical education for pediatric emergency skillsets, and ensure access to critical equipment and medicines. The PEHC-SAT pilot also demonstrated feasibility of deploying the tool in settings with limited internet access. The assessment length did not pose a barrier to completion, as participants submitted responses within the allocated one-month time frame. Blank responses were primarily limited to branching logic and were not concentrated at the end of the assessment, pointing away from survey fatigue.

The PEHC-SAT found higher frequency of triage tools being used at participating EDs (68.8%) compared to a 2001 study that assessed hospitals’ pediatric acute care in seven LMICs across Southeast Asia, the Caribbean, and Africa [28]. In that study, 14 out of 21 hospitals (66.7%) lacked an adequate system for triage [28]. There was also a lack of guidelines for standard assessment and treatment [28]. In contrast, the PEHC-SAT found pediatric clinical care protocols were available at 68.8% of participating EDs. The PEHC-SAT identified another strength: almost half of the EDs had pediatric champions, similar to rates in the 2013 NPRP survey [6]. In the US, pediatric champions had a significant impact on pediatric emergency preparedness scores, adherence to emergency care guidelines [6] and mortality [8, 29]. Similar to studies of EDs across Nigeria [30] and South Africa [5], the PEHC-SAT reflects challenges with access to pediatric resuscitation equipment, though the PEHC-SAT pilot showed overall higher rates of equipment availability. The larger proportion of tertiary care centers in this study may account for improved availability of pediatric equipment. Assessment methodology, specifically on-site equipment verification [5, 30], versus the PEHC-SAT self-reported responses may also account for these differences.

The PEHC-SAT operationalized pre-existing international standards and guidelines so EDs may identify gaps to advocate for resources and inform quality improvement efforts. EDs that are new to seeing pediatric patients may use the tool as a roadmap. The findings from this study demonstrate the feasibility of expanding the pilot to evaluate more EDs on a greater scale.

Limitations to this study include the sample size which limits defining PEHC trends in LMIC settings. Due to the sample size, there was no clear correlation between ED pediatric patient volume and availability of pediatric protocols, policies, or equipment. The geographic distribution and hospital type also limits the generalizability of the assessment tool results. While validation of the assessment tool is outside the scope of the study, lack of on-site verification of the assessment tool responses may impact the accuracy of the results. To mitigate potential challenges with self-reported assessment results, each participating ED received an individualized, descriptive PEHC-SAT report. The privately shared report provided PEHC descriptions and how to advance pediatric care rather than numerical scores (sample report in supplemental materials).

## Conclusions

This study reports the results from the pilot of a novel assessment tool evaluating pediatric emergency care capabilities in sub-Saharan Africa. The PEHC-SAT findings demonstrate that opportunities exist to improve emergency pediatric preparedness aligning EDs to international standards. The comprehensive PEHC-SAT is a valuable resource that identifies existing barriers to and gaps in emergency care for children, allowing EDs to utilize the data to prioritize resources. In future work, the PEHC-SAT will be revised and improved based on qualitative interview feedback received from participants. A future larger study with the revised tool would include representation from different regions and hospital classifications with the goal of validating the PEHC-SAT for use globally.

## Supplementary Information

Supplementary Material 1.

Supplementary Material 2.

Supplementary Material 3.

## Data Availability

The datasets used and/or analyzed during the current study are available from the corresponding author on reasonable request.

## Abbreviations

ED: Emergency Department
LMIC: Low- and middle-income country
NPRP: National Pediatric Readiness Project
IFEM: International Federation of Emergency Medicine
WHO: World Health Organization
ETAT: Emergency Triage Assessment and Treatment
AFEM: African Federation of Emergency Medicine
ECAT: Emergency Care Assessment Tool
PEHC-SAT: Pediatric Emergency Healthcare Capabilities Self-Assessment Tool
RCPCH: Royal College of Pediatrics and Child Health
HCW: healthcare worker
SOP: Standard Operating Procedure
BCHGHP: Boston Children’s Hospital Global Health Program
PALS: Pediatric Advanced Life Support
APLS: Advanced Pediatric Life Support
