# Supplementary material for: A novel tool for assessing pediatric emergency care in low- and middle-income countries: a pilot study
Source: Int J Emerg Med. 2025 Jan 16;18:15. doi: 10.1186/s12245-024-00802-2 (PMC11740608; doi:10.1186/s12245-024-00802-2)

Appendix 1:

Pediatric Emergency Healthcare Capabilities Self-Assessment Tool Outline by Domain


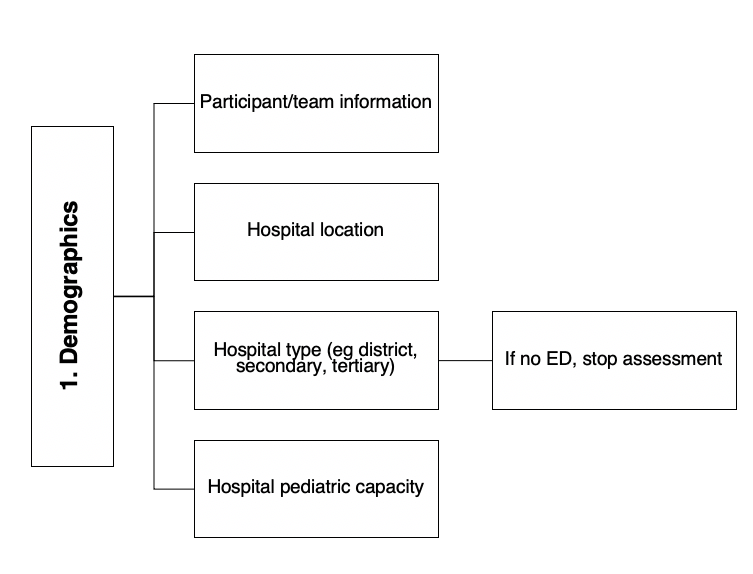


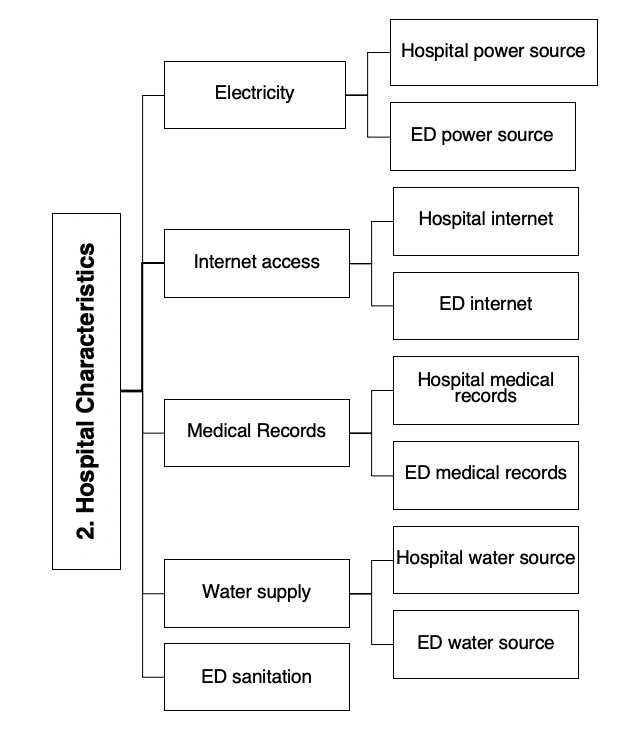


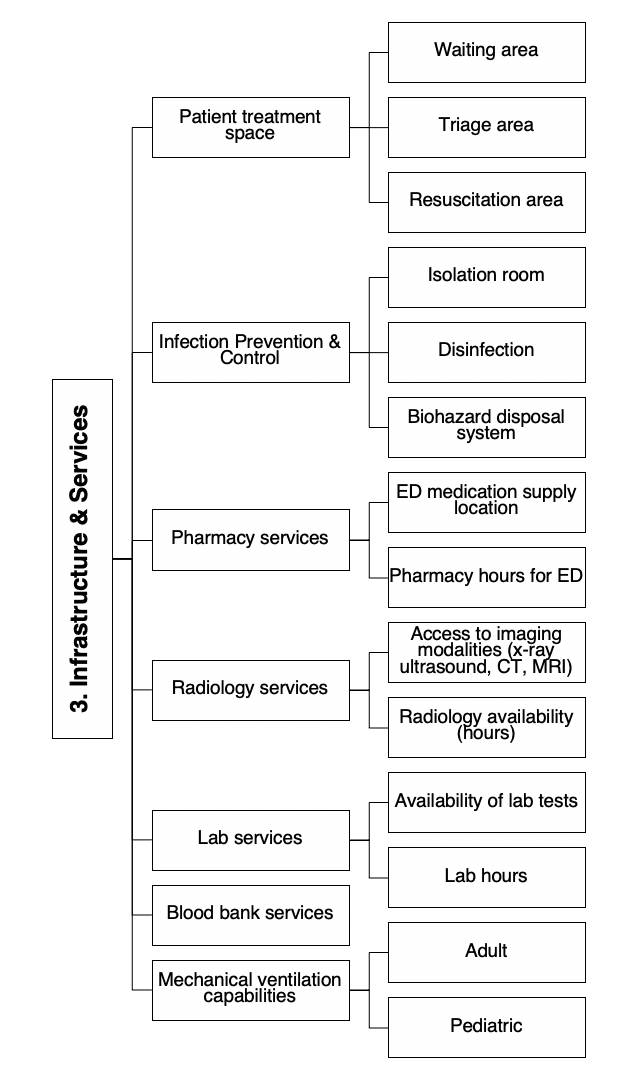


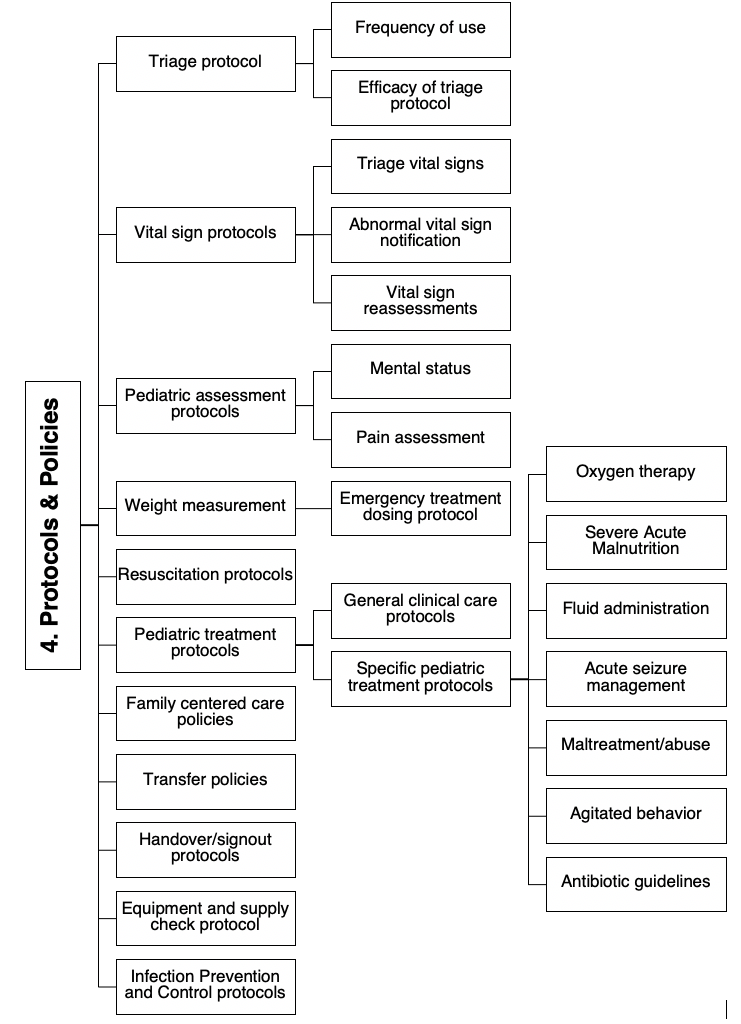


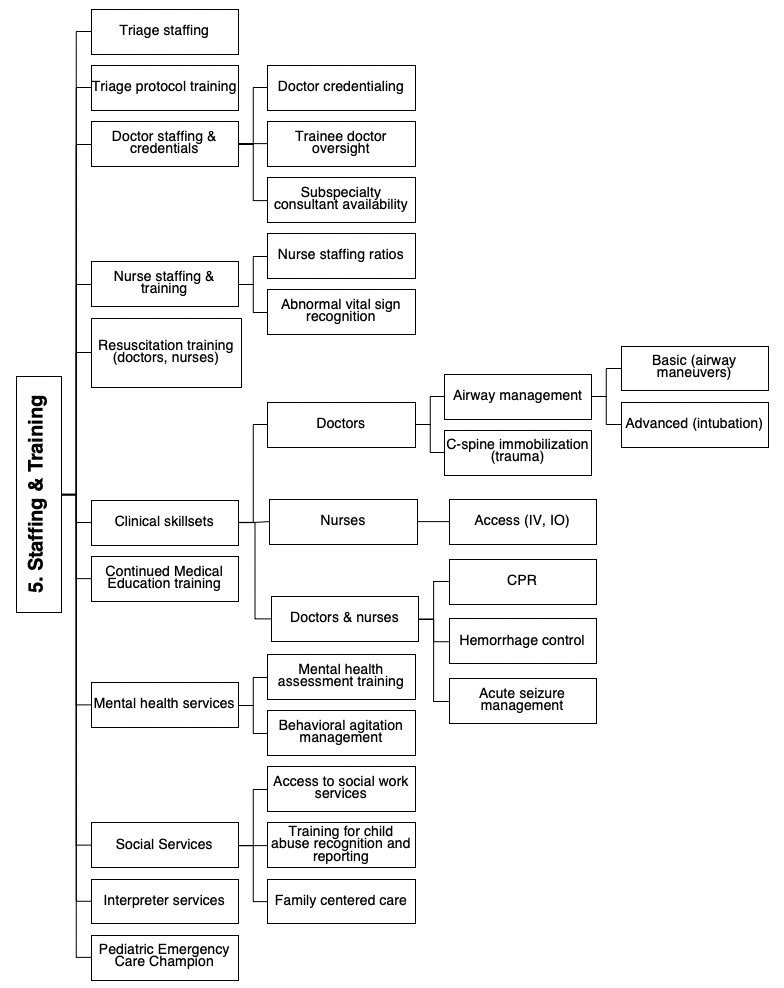


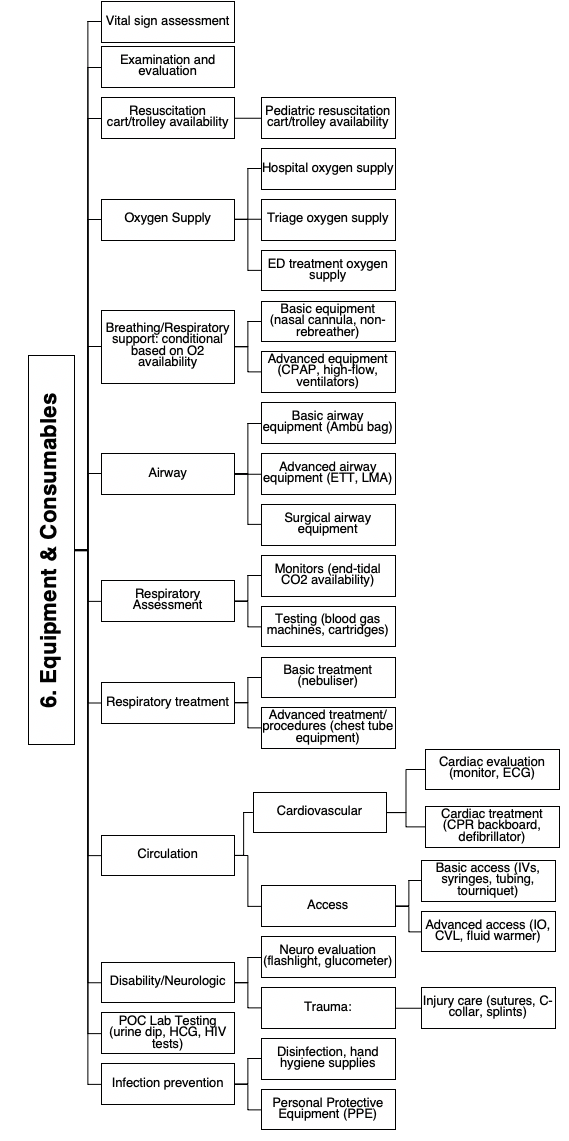


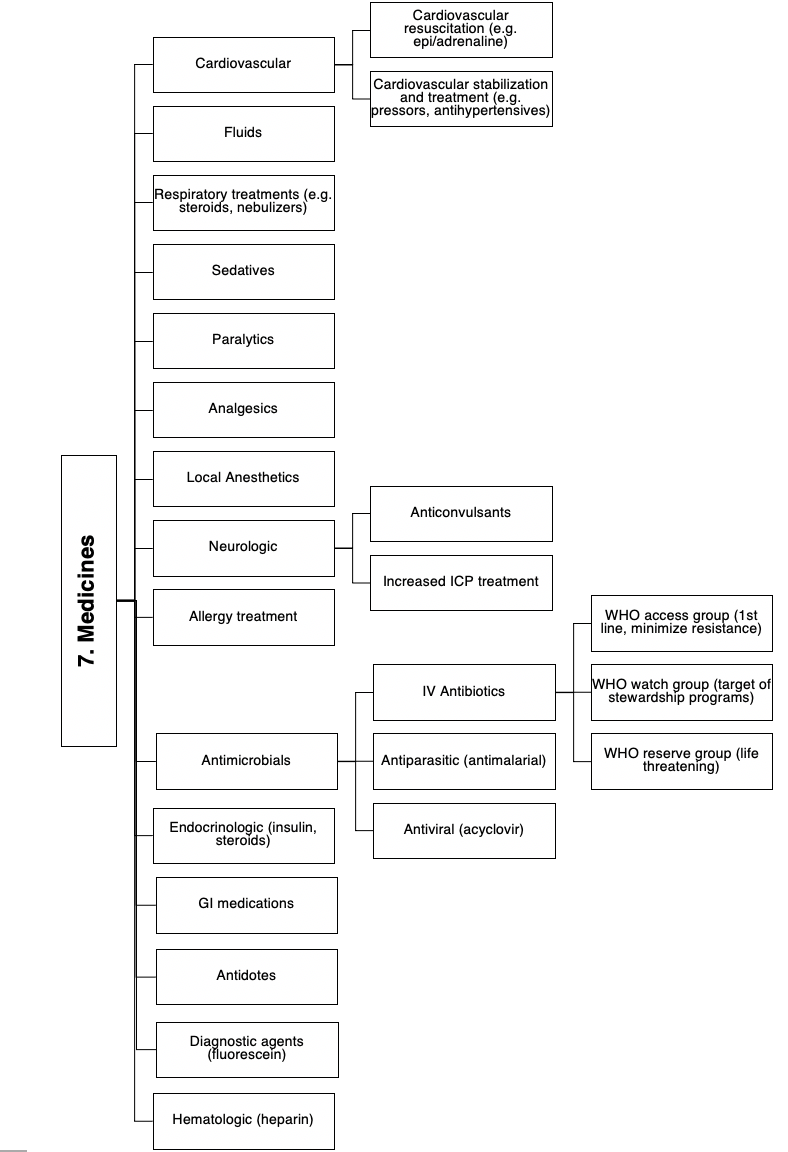

Supplement: Supplementary file 1 — Supplementary Material 1. [file 12245_2024_802_MOESM1_ESM.docx]
