# Supplementary material for: A novel tool for assessing pediatric emergency care in low- and middle-income countries: a pilot study
Source: Int J Emerg Med. 2025 Jan 16;18:15. doi: 10.1186/s12245-024-00802-2 (PMC11740608; doi:10.1186/s12245-024-00802-2)
Supplement: Supplementary file 2 — Supplementary Material 2. [file 12245_2024_802_MOESM2_ESM.docx]

**Pediatric Emergency Care Capacity Self-Assessment Tool**

**~User Guide~**

1. **Introduction:**

The purpose of this self-assessment tool is to help a hospital assess its pediatric emergency care capacity. Pediatric emergency care capacity refers to a hospital’s ability to triage, treat, stabilize, and resuscitate a pediatric patient for the first 24 hours after arrival. This tool has been created to make existing standards and guidelines easy to apply to your hospital environment. The tool’s sources include guidelines and standards from the International Federation of Emergency Medicine Pediatric Emergency Medicine Special Interest Group and the World Health Organization. This activity is a pilot of the tool. Your experience using the tool at your hospital will be critical to further refine and improve the tool.

1. **Purpose:**
   1. This user guide provides instructions to complete a Pediatric Emergency Care Capacity Self-Assessment using the tool developed.
   2. Piloting the tool will provide your hospital feedback on areas that it is meeting the needs for pediatric emergency care and areas that may require improvement or further investment.
2. **Definitions:**
   1. *Pediatric emergency care capacity*: a hospital’s ability to triage, treat, stabilize, and resuscitate a pediatric patient for the first 24 hours after presentation.
   2. *Participating site:* hospital piloting the self-assessment tool. Participating sites will not include outpatient clinics.
   3. *Participating site lead:* participant completing the self-assessment tool that serves as a leader to coordinate additional participants for the participating site.
   4. *Project team facilitator:* team member that introduces the assessment tool and provides support for tool completion
   5. *KoBoToolbox:* an open-source software used as the platform for the self-assessment. This software was chosen as it is the United Nations’ supported international standard for online data collection. [https://www.kobotoolbox.org/](mailto:sonia.jarrett@childrens.harvard.edu)
3. **Abbreviations:**

ED: emergency department

1. **Method for Use of the Tool:**

A project team facilitator will introduce the online self-assessment tool over a one hour video conference meeting. This meeting will provide a tutorial on how to use the online tool and give an overview of the methods that your hospital may choose to complete the tool. The participating site lead and any potential staff that may fill in the self-assessment tool should attend this meeting.

This Pediatric Emergency Care Capacity Self-Assessment Tool is designed so that hospitals may use it in the best way for their environment. After the introductory meeting, your hospital should select **one** method and complete the whole tool using that method. The assessment tool should be completed in one month.

**Methods for completing the tool:**

- 1. One person (the participating site lead) uses a laptop/tablet or paper print-out of the tool and fills the entirety of the tool themselves using their own knowledge and interviewing anyone else who may have input to answering the questions.
  2. A meeting is held (likely to take approximately 3 hours) bringing together the relevant parties to answer the questions (e.g. a physician and nurse working in pediatric ED care, a hospital administrator and a pharmacist). The tool is filled in together as a team on a laptop or tablet.
  3. The tool is filled in by multiple people at the hospital based on their role and its relevance to the sections of the tool. A tablet may be passed from one person to another to fill in the tool completely on one KoBoToolbox form or participating individuals complete their relevant sections of the tool on their own device at different times. The KoBoToolbox form cannot be filled in simultaneously by multiple people at once. Table 1 describes which hospital staff would be best to fill out each section of the tool.

The project team facilitator will check in weekly over email with the participating site lead to answer any questions about the assessment tool or troubleshoot problems with completion of the tool.

1. **Personnel Needed:**

Depends on the approach that the hospital chooses to take for their assessment. One person will be required to be the site lead to champion conducting the assessment at the hospital. Additional personnel may include:

- 1. Emergency Department Physician/Doctor
  2. Emergency Department Nurse
  3. Hospital administration point of contact
  4. Pharmacist

1. **Materials Needed:**

- Computer (Windows or Mac), tablet, or smart phone (iOS or Android acceptable)
- Web browser (any)
- Internet access: the online tool can be downloaded onto a device for offline completion (no specific software required)
- Paper for notetaking
- Pens or pencils for notetaking
- The online tool can also be printed and completed manually. The participating site lead could then enter it into KoBoToolbox or scan and email the paper form to [sonia.jarrett@childrens.harvard.edu](mailto:sonia.jarrett@childrens.harvard.edu) (note if printing: the tool will require 72 pages to print due to the formatting from electronic to paper)

1. **Steps to complete the pediatric care capacity tool:**
   1. Identify the participating site lead who will champion and lead completing the tool.
   2. Decide on which methodology (Section V above) you will use to conduct your assessment.
   3. Set up a shared KoBoToolbox account for your participating site on [kobotoolbox.org](https://kf.kobotoolbox.org/accounts/register/#/)
   4. Assemble the participants required to complete the Pediatric Emergency Care Capacity Self-Assessment Tool:
      1. The participating site will have one member identified to initiate the self-assessment. This participant may self-identify as the participating site lead, or they will identify another team member to serve in this role.
      2. The participating site lead will set up a date and time for meeting as a team, or individually with additional team members to complete the self-assessment
      3. The self-assessment tool contains 7 sections. Table 1 below gives an overview of each section and includes suggestions on who might be best to participate in completing that section of the tool. Some sections may require more multi-disciplinary teams to provide the requested information.

**Table 1**. Assessment tool section overview with suggested participants

| **Section** | **Description** | **Suggested participant role(s)** |
| --- | --- | --- |
| 1. Demographics | Collects information about the participants completing the self-assessment and the participating site. All information regarding the participants will be kept confidential | Doctor, nurse or nurse manager, hospital administrator with operations knowledge |
| 1. Hospital Characteristics | Obtains general information about the participating site including water supply, electricity, internet, and sanitation | Doctor, nurse or nurse manager, hospital administrator |
| 1. Infrastructure & Services | Assesses availability of a physical space designated for emergency care, associated diagnostic (e.g. laboratory and radiology) and treatment services (e.g. pharmacy and blood bank) | ED doctor, triage nurse, ED nurse or nurse manager, pharmacist, radiologist, laboratory technician, blood bank technician |
| 1. Protocols & Policies | Availability and use of pediatric-specific triage and resuscitation protocols and transfer policies | ED doctor, triage nurse, ED nurse or nurse manager, pharmacist |
| 1. Staffing & Training | Numbers and availability of qualified healthcare staff (doctors, nurses, support staff), their training background and continuing education | ED doctor, triage nurse, ED nurse or nurse manager, social workers, hospital administrator |
| 1. Equipment & Consumables | Availability and functionality of durable items (e.g. monitoring devices) and items intended for single-use in patient care (e.g. syringes, IV catheters) | ED doctor, triage nurse, ED nurse or nurse manager |
| 1. Medicines | Availability and accessibility of essential medicines | ED doctor, triage nurse, ED nurse or nurse manager, pharmacist |

- 1. To access the self-assessment tool online, follow this link https://ee.kobotoolbox.org/
  2. Open the assessment tool on your computer or tablet by opening it in a web browser. Once opened, it is downloaded and as long as you do not close the browser window it can be accessed on the device’s web browser online or offline. Progress can be saved using the save button when connected to Wi-Fi if the tool cannot be completed in one sitting.
  3. Complete the self-assessment tool using one of the three approaches described above in Section V. The anticipated time for completing the self-assessment is 2-3 hours. The self-assessment tool responses can only be submitted once. The goal is to provide the most accurate responses possible which may require consultation with the suggested participants listed in Table 1.
  4. Once completed, save and submit the self-assessment tool online through the KoBoToolbox platform.

**Figure 1.** Schematic Overview of Completing the Self-Assessment Tool:

**
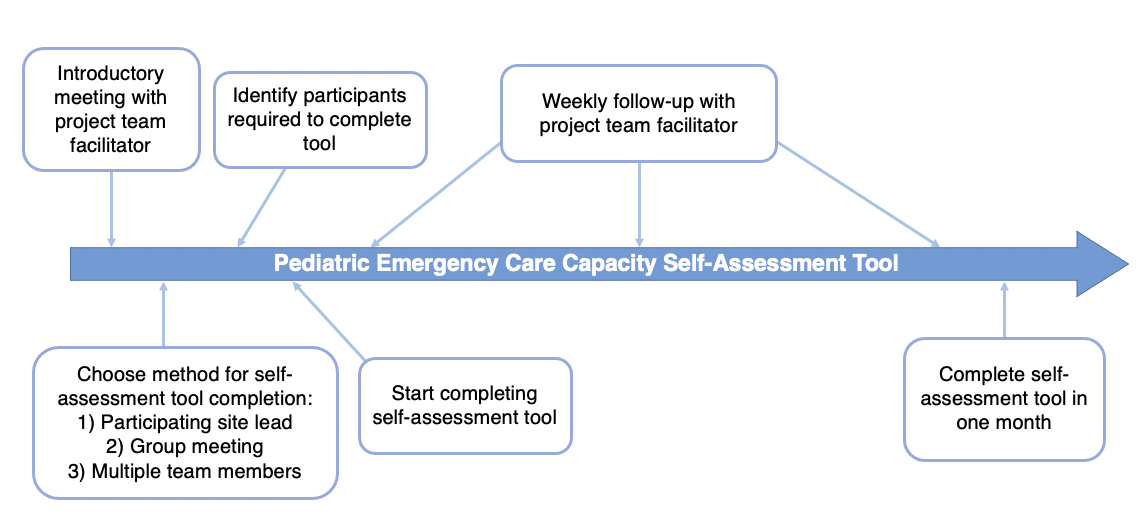
**

1. **Supplemental Materials:**

Schematic representation of each section of the Pediatric Emergency Care Capacity Self-Assessment Tool
