# Supplementary material for: A novel tool for assessing pediatric emergency care in low- and middle-income countries: a pilot study
Source: Int J Emerg Med. 2025 Jan 16;18:15. doi: 10.1186/s12245-024-00802-2 (PMC11740608; doi:10.1186/s12245-024-00802-2)
Supplement: Supplementary file 3 — Supplementary Material 3. [file 12245_2024_802_MOESM3_ESM.pdf]

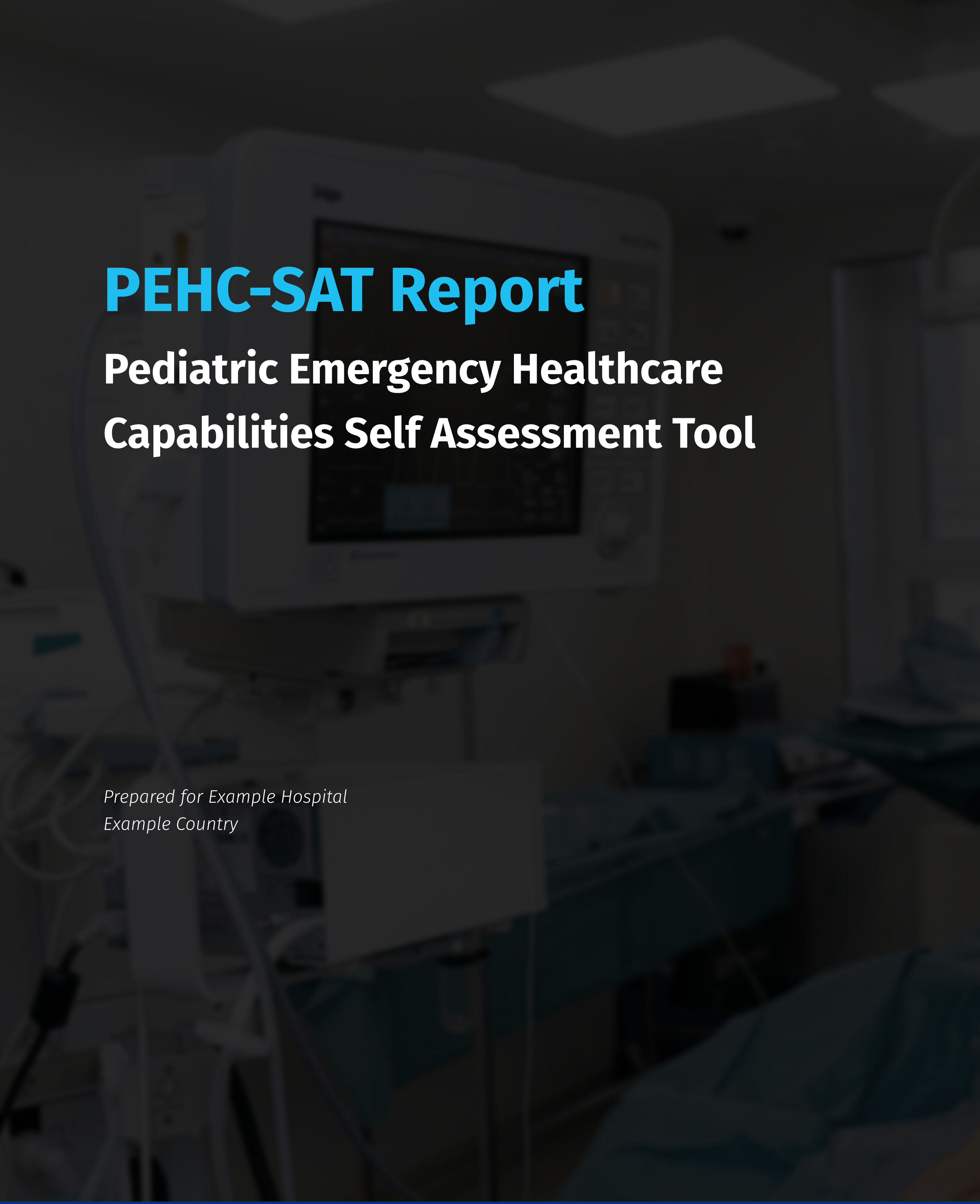

PEHC-SAT Report

Pediatric Emergency Healthcare Capabilities Self Assessment Tool

Prepared for Example Hospital
Example Country

Prepared by the Pediatric Preparedness Partners
in collaboration with Boston Children's Hospital

Boston Children's Hospital

Table of contents

- Introduction 3

Domains

- Emergency Department Characteristics & Infrastructure 6
- Pediatric Triage 9
- Pediatric Protocols & Policies 13
- Staffing & Training 22
- Ancillary Services 27
- Pediatric Resuscitation 32
- Pediatric Respiratory & Airway Support 37
- Pediatric Vascular Access 43
- Pediatric Trauma 46
- Infection Prevention & Control 48
- Medicines 51

Introduction

Providing emergency care to pediatric patients of all ages and sizes is not easy. Emergency care for children presents unique challenges to clinicians and hospitals. Children have specific anatomic, physiologic, developmental, and medical needs. Globally, the majority of children who seek emergency care present to general emergency departments (EDs) rather than specialized pediatric EDs. There remains significant variation in the provision of pediatric emergency care internationally.

To standardize the provision of effective emergency care to children, the World Health Organization (WHO), International Federation of Emergency Medicine (IFEM) Pediatric Emergency Medicine Special Interest Group (PEMSIG) have developed guidelines and consensus documents for the treatment and evaluation of pediatric emergency patients. In 2013, the United States (US) Emergency Medical Services for Children (EMSC) Program launched the ongoing quality improvement initiative, the National Pediatric Readiness Project (NPRP), to evaluate the readiness of US EDs to take care of pediatric emergency patients.

To evaluate pediatric emergency capabilities in low- and middle-income countries, a group of partners from Kenya, South Africa, Nigeria, and the United States developed a new online Pediatric Emergency Healthcare Capabilities Self-Assessment Tool (PEHC-SAT). The assessment tool is based on existing standards and guidelines set forth by the WHO, IFEM PEMSIG, and NPRP. Pediatric emergency care capabilities refer to a hospital's ability to triage, treat, and stabilize a pediatric patient (defined as newborn up to 18 years of age) for the first 24 hours after presentation, or prior to disposition. The PEHC-SAT is specifically designed for low- and middle-income country contexts. The assessment tool excludes outpatient and inpatient units.

We are grateful for your support and time to pilot the PEHC-SAT. This PEHC-SAT Report compiles the online assessment tool responses from ***your*** hospital and provides an overview of ***your*** hospital's capabilities to triage, treat, stabilize, and resuscitate pediatric emergency patients. This report provides a way to identify then prioritize your hospital's needs when it comes to providing the best pediatric emergency care possible.

How does the PEHC-SAT report work?

The report is divided into eleven domains that reflect key elements of pediatric emergency care:

1. Emergency Department Characteristics & Infrastructure
2. Pediatric Triage
3. Pediatric Protocols & Policies
4. Staffing & Training

5. Ancillary Services
6. Pediatric Resuscitation
7. Pediatric Respiratory & Airway Support
8. Pediatric Vascular Access
9. Pediatric Trauma
10. Infection Prevention & Control
11. Medicines

Each domain is divided into three progressive levels of pediatric emergency care capability which are: “Foundation,” “Intermediate,” and “Advanced.” The levels are characterized as follows:

- **Foundation:** refers to a core degree of pediatric emergency readiness that hospitals should be able to provide. Hospitals with a foundation level of preparedness may not have pediatric inpatient services.
- **Intermediate:** builds upon the foundation level with additional pediatric services and capabilities, including higher levels of support. Hospitals with an inpatient pediatric ward may have domains at the intermediate level.
- **Advanced:** continues to build upon both foundation and intermediate levels with more comprehensive pediatric services and specialty pediatric care. There may be pediatric intensive care capabilities at this hospital in addition to general pediatric inpatient wards.

To determine the domain level, there are “Components” and “Characteristics” that serve as criteria to assess different aspects of pediatric emergency care within the domain. For example, a “Component” would be “Radiology services.” This category is further divided into more specific “Characteristics” e.g. “X-ray services,” “Ultrasound services,” and “CT services.” A few “Components” and “Characteristics” may only have descriptions for Foundation and Advanced levels.

The report has categorized the completed PEHC-SAT responses into corresponding components and characteristics in each domain, highlighted in blue. The “overall level” is a compilation of all the PEHC-SAT results for that domain that summarizes the components and features. The reason for designating a domain level rather than a final domain score allows users to see what features may be needed to advance pediatric emergency care at their hospital. If data was missing from the assessment tool, that component or feature would be considered at the foundation level.

Each domain has a list of associated informational resources for pediatric emergency care. These resources include pre-existing guidelines and protocols as a reference and may be adapted to local contexts. The free, online OPENPediatrics resource (included in multiple domains) provides educational content about pediatric emergency care and a wide range of additional pediatric topics. OPENPediatrics requires a one-time registration for ***free*** access to its materials.

Recognizing that each hospital has its own unique strengths and needs, the PEHC-SAT report provides a framework for hospitals to assess their pediatric emergency care capabilities based on pre-existing international guidelines. This report is not meant to be a step-by-step guide to establish a pediatric ED or serve as the sole resource for pediatric emergency care development. The PEHC-SAT report serves as one additional resource for hospitals to use when adapting pediatric emergency care recommendations to their local needs and resource availability.

Questions and Feedback

Questions or feedback about the report are welcome and appreciated. Please email sonia.jarrett@childrens.harvard.edu to get in touch.

References

- Barrett J., Rodriguez M., Moegling, M., Chung, S. EMSC Innovation and Improvement Center. Disaster Domain Toolkit Subcommittee. (2022). Checklist of Essential Pediatric Domains and Considerations for Every Hospital's Disaster Policies.
- Remick K, Gausche-Hill M, Joseph MM, Brown K, Snow SK, Wright JL. Policy Statement: Pediatric readiness in the emergency department. *Am Coll Emerg Physicians*. 2018;142(5): 1-24. doi:10.1542/peds.2018-2459
- Institute of Medicine, Committee of the Future of Emergency Care in the US Health System. *Emergency Care for Children: Growing Pains*. Washington, DC: National Academies Press; 2007.
- AAP, ACEP. Care of children in the emergency department: Guidelines for preparedness. *Pediatrics*. 2001;107(4 I):777-781. doi:10.1542/peds.107.4.777
- Krug SE, Bojko T, Fein JA, et al. Joint policy statement - Guidelines for care of children in the emergency department. *Pediatrics*. 2009;124(4):1233-1243. doi:10.1542/peds.2009-1807
- NPRP. Pediatric Readiness Assessment and Scoring. https://emscimprovement.center/media/pediatric-readiness/pdfs/peds-ready-toolkit/Assessment_and_Scoring_CA2125.pdf?la=en
- World Health Organization (WHO). Updated guideline: paediatric emergency triage, assessment and treatment: care of critically-ill children. (2016) https://apps.who.int/iris/handle/10665/204463
- World Health Organization (WHO). Emergency Triage Assessment and Treatment (ETAT) course. (2005, February 20) https://www.who.int/publications/i/item/9241546875
- International Federation of Emergency Medicine. Standards of Care for Children in Emergency Departments Version 3. *Int Fed Emerg Med*. Published online 2019. https://www.ifem.cc/wp-content/uploads/2019/06/Standards-of-Care-for-Children-in-Emergency-Departments-V3-2019.pdf

Emergency Department Characteristics & Infrastructure

This domain assesses availability of utilities and resources in the emergency department (ED) that are relevant for medical care, including electricity source, water supply, methods of patient record-keeping and communication within hospital departments.

COMPONENTS & FEATURES		FOUNDATION	INTERMEDIATE	ADVANCED
Electricity source and internet access	Electricity source	Emergency department (ED) has access to one source of electricity without reliable back-up energy source	ED has access to electricity with back-up energy source	**ED has access to electricity with multiple back-up sources**
	Internet access	**Internet access is never available in the ED**	Internet access is available up to 50% of the time in the ED	Internet access is available more than 50% of the time in the ED
	Online medical resources	**Access to online current medical references is not available in the ED**	N/A	There is a device available in the ED with access to online current medical references (i.e. hospital-owned or personal desktop, laptop, or tablet)
Communications	Medical records: Individual records	The ED does not use individual patient charts for medical record keeping	N/A	**The ED uses individual patient charts for medical record keeping**
	Medical records: Retrievability of medical record	The medical record can only be used for one visit, the record cannot be retrieved for return visits	The medical record can be used for multiple visits and can be retrieved up to 50% of the time	**The medical record can be used for multiple visits and can be retrieved more than 50% of the time**
	Medical records: Electronic medical record	**No electronic medical record available**	Electronic medical record available and functioning up to 50% of the time	Electronic medical record available and functioning more than 50% of the time

COMPONENTS & FEATURES		FOUNDATION	INTERMEDIATE	ADVANCED
	Inter-department communication	Only one of the following methods is used for the ED to communicate with other hospital departments and the wards: speaking in person, personal cell phone, landline telephone, intercom, pager, or hospital provided handheld device	Two of the following methods for the ED to communicate with other hospital departments and the wards: speaking in person, personal cell phone, landline telephone, intercom, pager, hospital provided handheld device	Three or more of the following methods for the ED to communicate with other hospital departments and the wards: speaking in person, personal cell phone, landline telephone, intercom, pager, hospital provided handheld device
Water supply and plumbing	Water supply	Water supplied through rain water collection or bottled water	Water supplied through bore hole, well water source, surface water (e.g. river, lake, pond)	Water supplied through piped government/ municipal source
	Running water	Running water is never available in the ED	Running water is available up to 50% of the time in the ED	Running water is available more than 50% of the time in the ED
	Plumbing	No dedicated toilets for ED patients to use	Dedicated toilets are available for ED patients to use; flush toilets are functional up to 50% of the time	Dedicated toilets are available for ED patients to use; flush toilets are functional more than 50% of the time

Overall level for Emergency Department Characteristics & Infrastructure domain

FOUNDATION	INTERMEDIATE	ADVANCED
Electricity is available without a reliable back-source. Internet and online medical references are not available. Medical records are typically single-use only and not electronic. There is only one primary method of communication between hospital departments. Still water is available but there is no running water. There are no dedicated toilets for ED patients to use.	Electricity is available with at least one back-up energy source. Limited access to internet is available though there may not be access to current online medical references. Individual medical records, including an electronic version, can be used for multiple visits and retrieved up to half the time. There are at least two methods of communicating with other hospital departments and the wards. Running water is available and there are dedicated toilets that can be flushed up to half the time.	Electricity is available with multiple back-up sources. Internet is accessible and there are devices to access current online medical references online. Individual electronic medical records can be retrieved for multiple visits more than half of the time. There are three or more different methods for communicating between departments and wards in the hospital. Running water is available and dedicated toilets for ED patients can flush more than half of the time.

Resources

1. World Health Organization (WHO). (2023). Harmonized health facility assessment (HHFA): Combined questionnaire. https://www.who.int/publications/m/item/hhfa-combined-questionnaire-availability--readiness-and-management-and-finance-june-2023_core_only

2. Royal College of Paediatrics and Child Health (RCPCH). (2018, June). Chapter 2: Environment in emergency care settings. Facing the future: Standards for children in emergency care settings (pp. 19-23). https://www.rcpch.ac.uk/sites/default/files/2018-06/FTFEC%20Digital%20updated%20final.pdf

Pediatric Triage

Triage refers to the process of sorting sick children when they first arrive in hospital to categorize them based on severity of illness to prioritize their assessment and treatment. This domain assesses availability of triage space, triage protocols for pediatric patients, triage staffing, and equipment for triage assessments

COMPONENTS & FEATURES		FOUNDATION	INTERMEDIATE	ADVANCED
**Triage infrastructure**	Waiting space	The ED does not have a waiting area for pediatric patients	Pediatric patients share a common waiting area with adult ED patients	**Pediatric patients have a dedicated ED waiting area**
	Triage space	**The ED does not have a triage area for pediatric patients**	Pediatric patients share a common triage area with adult ED patients	Pediatric patients have a dedicated ED triage area
**Triage process (registration, payment)**		Pediatric patients must wait in order of arrival or for registration or payment more than 50% of the time before a first assessment is conducted to assess for emergency signs	Pediatric patients must wait in order of arrival or for registration or payment up to 50% of the time before a first assessment is conducted to assess for emergency signs	**Pediatric patients do not need to wait in order of arrival or for registration or payment before a first assessment is conducted**
**Triage protocol**		There is no formal triage tool or protocol for pediatric emergency patients, though a list of emergency signs may be used for pediatric patients who require immediate care	There is a triage tool or protocol for pediatric emergency patients and is utilized up to 50% of the time	**There is a triage tool or protocol for pediatric emergency patients that is utilized more than 50% of the time**
**Triage staffing**	Designated triage staff	**There is no designated staff member for triage**	N/A	There is at least one staff member designated to triage pediatric emergency care patients 24 hours a day
	Triage staff ratios	There is no designated staff for triage of pediatric emergency patients	**The average staff to patient ratio for triage is 1 staff member for greater than 10 patients**	The average staff to patient ratio for triage is 1 staff member for 1-10 patients

COMPONENTS & FEATURES		FOUNDATION	INTERMEDIATE	ADVANCED
**Triage staff training**	Triage staff background	Triage for pediatric emergency care patients is performed by nursing student or non-healthcare staff	**Triage for pediatric emergency care patients is performed by certified RNs for adults, general emergency medicine or emergency doctors**	Triage for pediatric emergency care patients is performed by certified RNs for pediatrics or pediatricians
	Triage training	**There is no specific triage training for staff**	N/A	Triage staff members receive triage training through a triage protocol or training program (country or hospital specific)
**Triage vital signs**	Triage vital sign measurement	Pediatric patients never receive an initial set of vital signs during triage which includes temperature, heart rate, respiratory rate, pulse oximetry	Pediatric patients receive an initial set of vital signs (including temperature, heart rate, respiratory rate, pulse oximetry) during triage up to 50% of the time	**Pediatric patients receive an initial set of vital signs (including temperature, heart rate, respiratory rate, pulse oximetry) during triage more than 50% of the time**
	Triage blood pressure measurement	Pediatric patients never receive an initial blood pressure measurement at triage	**Pediatric patients receive an initial blood pressure measurement during triage up to 50% of the time**	Pediatric patients receive an initial blood pressure measurement during triage more than 50% of the time
	Triage vitals communication	Pediatric patient triage vital signs are not recorded but passed on verbally to the treatment team	Pediatric patient vital signs are recorded on a separate piece of paper or on a patient's personal outpatient chart	**Pediatric patient vital signs are recorded on a newly created paper chart or in the patient's electronic health record**
**Vital measuring equipment**		The following vital sign monitoring equipment is unavailable in pediatric sizing: stethoscopes, thermometers, pulse oximetry, blood pressure cuffs	Of the following vital sign monitoring equipment, up to 50% of the following are available in pediatric sizing: stethoscopes, pulse oximetry, thermometers, blood pressure cuffs	**Of the following vital sign monitoring equipment, more than 50% are available in pediatric sizing: stethoscopes, pulse oximetry, thermometers, blood pressure cuffs**

COMPONENTS & FEATURES		FOUNDATION	INTERMEDIATE	ADVANCED
Patient assessment equipment		Of the following examination equipment, otoscopes, ophthalmoscopes, and examination lights are not available in the ED for pediatric patients	One of the following pieces of examination equipment is available for pediatric ED patient evaluation: otoscope, ophthalmoscope, or examination light	All of the following pieces of examination equipment are available for pediatric ED patient evaluation: otoscope, ophthalmoscope, or examination light
Triage weight	Triage weight protocol	Pediatric patients are not weighed in kilograms prior to treatment in the ED	N/A	Stable pediatric patients are weighed in kilograms prior to treatment in the ED
	Pediatric weight documentation	Patient weights are not recorded or verbally passed on to treatment team members	Patient weights are recorded on paper either on a separate piece of paper, patient's personal chart, or ED-specific chart	Patient weights are recorded in the patient's electronic health record
	Pediatric weighing equipment	There are no weighing scales (in kilograms) available for pediatric patients	There are weighing scales available with 1 for more than every 11 pediatric patients	There are weighing scales available with 1 for every 10 or fewer pediatric patients

Overall level for Pediatric Triage domain

FOUNDATION	INTERMEDIATE	ADVANCED
The ED does not have a triage or waiting area for pediatric patients. Emergency signs may be used for initial assessment rather than a formal triage protocol. Pediatric patients may need to wait in order of arrival more than half of the time before an initial assessment. Triage may be conducted by non-healthcare staff or nursing students without specific triage training. Pediatric vital sign evaluation equipment may not be available and patients do not receive an initial set of vital signs. Weighing scales (in kilograms) may not be available and stable pediatric patients may not be weighed before receiving treatment.	The ED may have a common waiting and triage area for pediatric and adult patients. A formal triage protocol may be used up to half of the time. Pediatric patients wait in order of arrival less than half of the time before an initial assessment. Designated staff such as certified RNs for adults or general emergency medicine, or emergency doctors conduct triage. Triage staff receive training through a protocol or program. Pediatric patients receive initial vital signs up to half of the time recorded on either a paper or the patient chart. Vital sign evaluation equipment and patient exam equipment is available up to half of the time. Stable pediatric patients are weighed on scales (in kilograms) prior to treatment in the ED.	The ED likely has a dedicated waiting and triage area for pediatric patients. A formal triage protocol is used more than half of the time. Pediatric patients do not wait in order of arrival before an initial assessment. Designated staff such as certified pediatric RNs or pediatricians conduct triage. Triage staff receive training through a protocol or program. Pediatric patients receive initial vital signs more than half of the time. The vitals are recorded on the patient chart which may be electronic. Vital sign evaluation equipment and patient exam equipment is available more than half of the time. Stable pediatric patients are weighed on scales (in kilograms) prior to treatment in the ED.

Resources

1. WHO. (2005). Emergency triage assessment and treatment (ETAT): Manual for participants. https://www.who.int/publications/i/item/9241546875
2. WHO. (2016). Updated guideline: Paediatric emergency triage, assessment and treatment. Care of critically ill children. https://www.who.int/publications/i/item/9789241510219
3. Emergency Medicine Society of South Africa. (2019, November). The South African Triage Scale (SATS). https://emssa.org.za/special-interest-groups/the-south-african-triage-scale-sats/
4. Gilboy N, Tanabe T, Travers D, Rosenau AM. (2012). Emergency Severity Index (ESI): A triage tool for Emergency Department Care. Emergency Severity Index (ESI): A Triage Tool for Emergency Department Care, Version 4. Implementation Handbook 2012 Edition. Agency for Healthcare Research and Quality (AHRQ). https://media.emscimprovement.center/documents/ESI_Handbook2125.pdf

Pediatric Protocols & Policies

This domain evaluates the availability of protocols and policies directed toward the assessment and treatment of pediatric emergency patients. Protocols covered include initial pediatric patient assessment, specific pediatric clinical conditions, child maltreatment/abuse, pediatric mental health disorders. This domain also reviews policies with a focus on family centered care, the principle that families are a pediatric patient's primary source of support. Protocols for the signout of pediatric emergency patients between ED shifts and transfer between hospitals (facilities) are reviewed. Staff training for these specific policies and protocols are included in this domain. General staffing and training is reviewed in the next domain. For certain protocols, only Foundation and Advanced levels apply due to the binary nature of protocol availability.

COMPONENTS & FEATURES		FOUNDATION	INTERMEDIATE	ADVANCED
Pediatric vital signs protocols	Abnormal vital signs	There is no process in place for notifying doctors about abnormal pediatric patient vital signs	**Of the following methods, there is one process in place for notifying doctors about abnormal pediatric vital signs: verbally discussing with the treating provider; writing on a separate paper, not the medical record, to give to the treating provider; marking on a patient's paper medical record; indicating on the electronic medical record; recording on a bedside chart**	Of the following methods, there is more than one process in place for notifying doctors about abnormal pediatric vital signs: verbally discussing with the treating provider; writing on a separate paper, not the medical record, to give to the treating provider; marking on a patient's paper medical record; indicating on the electronic medical record; recording on a bedside chart
	Recognition of abnormal vitals	Nurses do not receive training on recognition of abnormal pediatric vital signs by age	N/A	**Nurses receive training on recognition of abnormal pediatric vital signs by age**
	Vital sign reassessments	There is no protocol for vital sign reassessment	N/A	**There is a protocol in place for vital sign reassessments**

COMPONENTS & FEATURES		FOUNDATION	INTERMEDIATE	ADVANCED
Pediatric patient assessment	Level of consciousness	There are no protocols in place for assessing pediatric patients' level of consciousness	All pediatric patients receive an assessment for level of consciousness with a visual assessment (no formal method)	All pediatric patients receive an assessment for level of consciousness with a formal method (AVPU - Alert, Voice, Pain, Unresponsiveness scale, Glasgow Coma Scale (GCS), Modified GCS, Blantyre Coma Scale)
	Pain Assessment	There are no protocols in place for assessing pediatric patients' level of pain	All pediatric patients receive an assessment for level of pain through observation of the patient's behavior or verbal self report (no formal method)	All pediatric patients receive an assessment for level of pain with a formal method (pictoral representation with the Wong-Baker Scale, or a numeric scale)
General pediatric clinical protocols		The ED does not use clinical care protocols designed specifically for pediatric patients	N/A	The ED uses clinical care protocols designed specifically for pediatric patients, for example, the World Health Organization Emergency Triage Assessment Tool (WHO ETAT), hospital-specific pediatric clinical protocols, publicly available online pediatric clinical protocols, WHO Hospital Care for Children, Oxford Handbook of Emergency Medicine

COMPONENTS & FEATURES		FOUNDATION	INTERMEDIATE	ADVANCED
Condition-specific pediatric clinical protocols		The ED does not use medical condition-specific clinical protocols to guide treatment for pediatric patients. Protocol examples include initiation of oxygen therapy for children with respiratory distress, severe acute malnutrition (SAM); IV fluid resuscitation in patients with signs of imapired circulation with and without SAM; acute seizure control; acute gastroenteritis; asthma exacerbation; diabetic ketoacidosis, severe malaria; sickle cell crises	The ED uses up to 3 different medical condition-specific clinical protocols to guide treatment for pediatric patients. Protocol examples include initiation of oxygen therapy for children with respiratory distress, severe acute malnutrition (SAM); IV fluid resuscitation in patients with signs of imapired circulation with and without SAM; acute seizure control; acute gastroenteritis; asthma exacerbation; diabetic ketoacidosis, severe malaria; sickle cell crises	The ED uses greater than 3 medical condition-specific clinical protocols to guide treatment for pediatric patients. Protocol examples include initiation of oxygen therapy for children with respiratory distress, severe acute malnutrition (SAM); IV fluid resuscitation in patients with signs of imapired circulation with and without SAM; acute seizure control; acute gastroenteritis; asthma exacerbation; diabetic ketoacidosis, severe malaria; sickle cell crises
Severe Acute Malnutrition (SAM) management	SAM protocol	There is not a protocol for recognizing pediatric patients with SAM	N/A	There is a protocol for recognizing pediatric patients with SAM, examples include Z-score and Mid-Upper Arm Circumference (MUAC)
	SAM training	Doctors and nurses are not trained in how to assess for malnutrition in pediatric patients	N/A	Doctors and nurses are trained in how to assess for malnutrition in pediatric patients
Child maltreatment/ abuse protocol	Child abuse reporting	The ED does not have a protocol for reporting suspected child maltreatment/ abuse	N/A	The ED has a protocol for reporting suspected child maltreatment/abuse
	Detection of child abuse	Doctors and nurses in the ED do not receive training in recognition of child abuse	N/A	Doctors and nurses in the ED receive training in recognition of child abuse

COMPONENTS & FEATURES		FOUNDATION	INTERMEDIATE	ADVANCED
	Training to report child abuse	Doctors and nurses in the ED do not receive training in reporting child abuse to authorities	N/A	Doctors and nurses in the ED receive training in recognition of child abuse
**Pediatric mental health protocols**	Mental health screening	The ED does not have a protocol for suicide screening in pediatric emergency patients	N/A	The ED has a protocol for suicide screening in pediatric emergency patients
	Behavioral health protocols	The ED does not have a protocol for pediatric patients presenting with acute mental health complaints	N/A	The ED has a protocol for pediatric patients presenting with acute mental health complaints. Protocol examples include guidance for treatment of pediatric patients with acute agitation or suicidality
	Mental health assessment training	Doctors and nurses do not receive training on the assessment of children's acute mental health conditions (e.g. suicidality, agitated behavior, psychosis)	N/A	Doctors and nurses receive training on the assessment of children's acute mental health conditions (e.g. suicidality, agitated behavior, psychosis)
	Restraint training	Doctors and nurses do not receive training on the use of restraints for agitated pediatric patients	N/A	Doctors and nurses receive training on the appropriate use of restraints for agitated pediatric patients

COMPONENTS & FEATURES		FOUNDATION	INTERMEDIATE	ADVANCED
Family Centered Care Protocols	Family Centered Care methods	The ED does not involve families in pediatric emergency patient care	The ED uses up to 3 different methods for promoting family centered care. Examples of these family centered methods include the following: 1) involving families or caregivers in patient care decision-making; 2) involving families or caregivers in medication safety processes by having them identify the patient; 3) family/caregiver presence during all aspects of emergency care, including resuscitation; 4) education of the patient, family, and caregivers on treatment plan and disposition; 5) allowing families to see and hold their deceased child	The ED greater than 3 different methods for promoting family centered care. Examples of these family centered methods include the following: 1) involving families or caregivers in patient care decision-making; 2) involving families or caregivers in medication safety processes by having them identify the patient; 3) family/caregiver presence during all aspects of emergency care, including resuscitation; 4) education of the patient, family, and caregivers on treatment plan and disposition; 5) allowing families to see and hold their deceased child
	Family/ caregiver presence	There is never sufficient space in the ED to accommodate a parent or caregiver to be with the pediatric patient at all times	There is sufficient space available up to 50% of the time in the ED to accommodate a parent or caregiver to be with the pediatric patient at all times	There is sufficient space available more than 50% of the time in the ED to accommodate a parent or caregiver to be with the pediatric patient at all times
	Decreasing stress for pediatric patients	There are no ways to separate pediatric patients from distressing sights and sounds of other patients in the ED	There is one method for separating pediatric pateints from distressing sights and sounds in the ED, examples include a portable screen, curtains between patients, individual rooms with doors	There is more than one method for separating pediatric pateints from distressing sights and sounds in the ED, examples include a portable screen, curtains between patients, individual rooms with doors

COMPONENTS & FEATURES		FOUNDATION	INTERMEDIATE	ADVANCED
	Pediatric patient death protocol	There is not a protocol regarding what happens after the death of a pediatric patient in the ED	N/A	**There is a protocol regarding what happens after the death of a pediatric patient in the ED (examples include communicating the death of a child, support of the bereaved with services including social work)**
	Staff training for pediatric deaths	**Doctors and nurses working in the ED do not receive training in supporting parents or caregivers in response to a pediatric patient death**	N/A	Doctors and nurses working in the ED receive training in supporting parents or caregivers in response to a pediatric patient death
Patient transfer protocols	Handover at shift change	Between shifts there is no handover/signout process for transfer of care of pediatric patients from one provider to another in the ED	N/A	**Between shifts there is a handover/signout process for transfer of care of pediatric patients from one provider to another in the ED**
	Transfer out of hospital policy	**The ED does not have a guideline to initiate transfer of pediatric patients to another facility**	N/A	The ED has a guideline to initiate transfer of pediatric patients to another facility, including the roles and responsibilies of the referring hospital and referral center

Overall level for Pediatric Protocols & Policies domain

FOUNDATION	INTERMEDIATE	ADVANCED
Protocols are not available for the following aspects of pediatric assessment: abnormal vital sign communication; vital sign reassessment; level of consciousness; level of pain. There are no condition-specific clinical protocols for pediatric patients. Protocols are not available for the reporting of child maltreatment/abuse or screening for or management of acute pediatric mental health complaints. Family centered care is not possible due to insufficient space and protocols are not available. Between shifts there is no sign-out process between ED providers. There is no guideline for transfers of pediatric patients to another facility.	Some pediatric emergency clinical protocols are available including: a method of communicating abnormal pediatric vital signs; visual assessments of pediatric patients' level of consciousness or pain. There are up to three condition-specific protocols to guide treatment of pediatric patients. There may be protocols for the reporting child maltreatment/abuse and screening for acute pediatric mental health complaints. Doctors and/or nurses do not receive training for reporting child maltreatment/abuse or the assessment and management of mental health conditions. There are up to three methods for promoting family centered care. Between shifts there may be a sign-out process between ED providers. There may be a guideline for transfers of pediatric patients to another facility.	The following pediatric emergency protocols are available including more than one method for communicating abnormal pediatric vital signs; formal assessment of pediatric patients' level of consciousness and pain with a scoring system or scale. General pediatric clinical care protocols are used, e.g. WHO ETAT, WHO Hospital Care for Children, Oxford handbook of Emergency Medicine. There may be more than three condition-specific protocols to guide treatment of pediatric patients. There are protocols for the reporting of child maltreatment/abuse. There may be protocols for screening for and management of acute pediatric mental health complaints. There are more than three methods for promoting family centered care. Between shifts there is a sign-out process between ED providers. There may be a guideline for transfer of pediatric patients to another facility.

Resources

The pediatric clinical protocols included from the Emergency Medical Services for Children (EMSC) Innovation and Improvement Center (EIIC), the Royal College of Paediatrics and Child Health (RCPCH), and the Children's Hospital of Philadelphia (CHOP) are listed only as examples for reference and do not replace any local guidelines or policies. Each hospital and country setting may have their own different pediatric clinical protocols. Please continue to utilize the clinical protocols designed for your hospital. Any OPENPediatrics video resources may be accessed for free by registering for an account at https://www.openpediatrics.org/

1. WHO. (2005). Emergency triage assessment and treatment (ETAT): Manual for participants. https://www.who.int/publications/i/item/9241546875

2. IFEM. (2019). Chapter twelve: Policies, procedures & guidelines. Standards of care for children in emergency departments (pp. 71-74). https://assets.nationbuilder.com/ifem/pages/286/attachments/original/1650673696/Standards-of-Care-for-Children-in-Emergency-Departments-V3-April-2019.pdf?1650673696

3. Hayes L., Kielian A., Sham L., Moye B., Huth K., Patel A. (2020, December). Management of pediatric status epilepticus in resource-limited settings [Video]. OPENPediatrics. https://learn.openpediatrics.org/learn/course/internal/view/elearning/4899/Management-of-Pediatric-Status-Epilepticus-in-Resource-Limited-Settings.

4. Emergency Medical Services for Children Innovation and Improvement Center (EIIC). (n.d.). Condition-Specific Resources: Pediatric Education and Advocacy Kits (PEAKs). https://emscimprovement.center/education-and-resources/peak/

5. EIIC-Translating Emergency Knowledge for Kids (TREKK). (2022, April). Pediatric status epilepticus algorithm. https://emscimprovement.center/education-and-resources/peak/peak-status-epilepticus/trekk-eiic-pediatric-status-epilepticus-practice-guideline/

6. Morrissette, M., Newton, A., Freedman, S., Katz, L. (2021, September). Bottom line recommendations: Suicidal risk screening and assessment practice guideline. EIIC-TREKK. https://emscimprovement.center/education-and-resources/peak/pediatric-suicide-screening-mental-health/eiic-trekk-bottom-line-recommendation-suicidal-risk-screening-and-assessment-practice-guideline/

7. Ali, S., Chumpitazi, C., Drendel, A., Poonai, Naveen. (2023, June). Bottom line recommendations: Pain treatment. EIIC-TREKK. https://emscimprovement.center/education-and-resources/peak/peak-pediatric-pain/bottom-line-recommendation-pediatric-pain-treatment/

8. EIIC-TREKK. (2022, April). Care of the agitated patient. https://media.emscimprovement.center/documents/EMS220128_Agitation24x36_240102.pdf

9. Auerbach, M., Coombs, C., Lindberg, D., Magaña, J., Ornstein, A., Sharma, S., Tiyyagura, G. (2023, October). Bottom line recommendations: Physical child abuse. EIIC-TREKK. https://media.emscimprovement.center/documents/EMS230908_ChildAbuseBLRUPATE_231019_w2igJHI.pdf

10. RCPCH. (2018, June). Facing the future: Standards for children in emergency care. https://www.rcpch.ac.uk/sites/default/files/2018-06/FTFEC%20Digital%20updated%20final.pdf

11. RCPCH. (2018, September). Asthma: Quality standards. National Institute for Health and Care Excellence (NICE). https://www.nice.org.uk/guidance/qs25

12. RCPCH. (2016, June). Bronchiolitis in children: Quality standards. NICE. https://www.nice.org.uk/guidance/qs122

13. RCPCH. (2023, December). Epilepsies in children, young people and adults. Quality standard. NICE. https://www.nice.org.uk/guidance/qs211

14. RCPCH. (2016, February). Fractures (non-complex): assessment and management. NICE. https://www.nice.org.uk/guidance/ng38

Staffing & Training

This domain assesses availability of qualified healthcare staff that treat pediatric patients in the ED including doctors, nurses, subspecialists, and support staff. The training background and continuing education of these healthcare staff are covered in this domain. Some staffing characteristics only Foundation and Advanced levels apply due to the binary nature of staff availability.

COMPONENTS & FEATURES		FOUNDATION	INTERMEDIATE	ADVANCED
Doctor Staffing	Dedicated ED staffing	**There is not a doctor that is dedicated to working specifically in the ED 24/7**	N/A	There is a doctor that is dedicated to working specifically in the ED 24/7
	Doctor staffing ratios	On average, the approximate doctor to pediatric patient ratio in the ED is 1 doctor to greater than 10 patients	**On average, the approximate doctor to pediatric patient ratio in the ED is 1 doctor to 5-10 patients**	On average, the approximate doctor to pediatric patient ratio in the ED is 1 doctor to 1-4 patients
	Consultant location	When providing the majority of supervision of trainee doctors or medical officers, consultants (attendings) are on call and reachable by phone but do not come into the hospital or ED	When providing the majority of supervision of trainee doctors or medical officers, consultants (attendings) are on call near to the hospital (<30 minutes away) or on call inside the hospital	**When providing the majority of supervision of trainee doctors or medical officers, consultants (attendings) are physically located in the ED (or no trainees so not applicable)**
	Consultant staffing availability	Consultants are available to supervise trainee doctors or medical officers less than 8 hours a day	**Consultants are available to supervise trainee doctors or medical officers between 8-23 hours a day**	Consultants are available to supervise trainee doctors or medical officers 24 hours a day

COMPONENTS & FEATURES		FOUNDATION	INTERMEDIATE	ADVANCED
Training for doctors	Doctor specialty training background	Doctors with the following type of training background take care of pediatric patients in the ED: General/Family Practitioner certified and Internal Medicine certified	**Doctors with the following type of training background take care of pediatric patients in the ED: Pediatrics certified, Emergency Medicine certified**	Doctors with the following type of training background take care of pediatric patients in the ED: Pediatric Emergency Medicine certified
	Pediatrics credentialing for doctors	Pediatrics-specific credentialing is not required of doctors taking care of pediatric patients in the ED	**One pediatrics-specific credentialing program is required of doctors taking care of pediatric patients in the ED. Examples of these programs include: specialty credentialing (e.g. Pediatrics, Emergency, etc.) according to country guidelines; hospital specific competency evaluations (e.g. sedation, patient safety, etc.); continuing education requirements in pediatric emergency care (e.g. specific number of conferences, lectures per year in pediatrics, etc.); international standard programs (e.g. PALS, APLS, NRP, Helping Babies Breath)**	More than one pediatrics-specific credentialing program is required of doctors taking care of pediatric patients in the ED. Examples of these programs include: specialty credentialing (e.g. Pediatrics, Emergency, etc.) according to country guidelines; hospital specific competency evaluations (e.g. sedation, patient safety, etc.); continuing education requirements in pediatric emergency care (e.g. specific number of conferences, lectures per year in pediatrics, etc.); international standard programs (e.g. PALS, APLS, NRP, Helping Babies Breath)
Subspecialist staffing	Subspecialist consultation services	**There are no subspecialist consultants available for pediatric patients in the ED**	There are up to 3 different subspecialties available for consultation for pediatric patients in the ED. Subspecialties include: Surgery, Orthopedics, Cardiology, Neurology, Infectious Disease, other surgical subspecialties (e.g. Neurosurgery, Urology), other medical subspecialties (e.g. Endocrinology, Nephrology)	There are more than 3 different subspecialties available for consultation for pediatric patients in the ED. Subspecialties include: Surgery, Orthopedics, Cardiology, Neurology, Infectious Disease, other surgical subspecialties (e.g. Neurosurgery, Urology), other medical subspecialties (e.g. Endocrinology, Nephrology)

COMPONENTS & FEATURES		FOUNDATION	INTERMEDIATE	ADVANCED
	Subspecialist consultation location	Subspecialists are available by phone but do not come in to the ED to evaluate the pediatric patient	Subspecialists come for consultation when called by phone which may take longer than 30 minutes	**Subspecialists come for consultation when called by phone and typically take less than 30 minutes**
	Subspecialist consultant availability	Pediatric subspecialists are not available at any time of day	**Pediatric subspecialist consultants (surgery, orthopedics, cardiology, toxicology) are available on work days (Mondays - Fridays) between 8-23 hours a day**	Pediatric subspecialist consultants (surgery, orthopedics, cardiology, toxicology) are available on work days (Monday - Fridays) for 24 hours a day
Nursing staff ratios		On average, the approximate nurse to pediatric patient ratio in the ED is 1 nurse to greater than 10 patients	**On average, the approximate nurse to pediatric patient ratio in the ED is 1 nurse for every 5-10 patients**	On average, the approximate doctor to pediatric patient ratio in the ED is 1 nurse for every 1-4 patients
Continuing medical education (CME) for healthcare staff	CME for doctors	**Doctors do not receive continuing medical education (CME) opportunities**	Doctors receive CME opportunities annually	Doctors receive CME opportunities weekly to monthly
	CME for nurses	**Nurses do not receive CME opportunities**	Nurses receive CME opportunities annually	Nurses receive CME opportunities weekly to monthly
	CME for support staff (respiratory therapists, pharmacists)	**Support staff (e.g. respiratory therapists, pharmacists) do not receive CME opportunities**	Support staff (e.g. respiratory therapists, pharmacists) receive CME opportunities annually	Support staff (e.g. respiratory therapists, pharmacists) receive CME opportunities weekly to monthly
Pediatric champion		There is no lead doctor or lead nurse "pediatric champion" to raise awareness of specific needs of pediatric patients in the ED	There is a lead doctor or lead nurse "pediatric champion" to raise awareness of specific needs of pediatric patients in the ED	**There are both lead doctor and lead nurse "pediatric champions" to raise awareness of specific needs of pediatric patients in the ED**

COMPONENTS & FEATURES	FOUNDATION	INTERMEDIATE	ADVANCED
Equipment location training	ED staff do not receive training on location of equipment and medications	ED staff receive training on location of equipment and medications via either of the following methods: verbal overview of location of equipment and medications given by an experienced staff member while outside of the ED or a written description of location of equipment and medications	ED staff receive training on location of equipment and medications via physically walking through ED with an experienced staff member showing locations of equipment and medications

Overall level for Staffing & Training domain

FOUNDATION	INTERMEDIATE	ADVANCED
There is no doctor dedicated to working in the ED 24/7. Doctors see a volume of patients with a ratio of approximately 1 doctor to greater than 10 patients. Consultants (attendings) are not physically located in the ED but are reachable by phone. Consultant supervision is available less than 8 hours a day. Doctors come from a training background including general/ family practitioners and internal medicine. Pediatrics-specific credentialing is not required. Subspecialist consultants are available by phone but not able to see patients in the ED. Nurses see a volume of patients with a ratio of 1 nurse to greater than 10 patients. The doctors, nurses, support staff (respiratory therapists, pharmacists) do not receive CME opportunities. There are no doctor or nurse "pediatric champions."	There may not be a specific doctor designated to work in the ED 24/7. Doctors see a volume of patients with a ratio of approximately 1 doctor to fewer than 10 patients. Consultants are usually on call near the hospital or within the hospital. They may be available to supervise trainee doctors or medical officers 8 to 23 hours a day. Doctors may come from a training background including pediatrics and emergency medicine. Pediatric credentialing may be required. Up to 3 different subspecialities are available for consultation including surgical or medical subspecialties. Subspecialists may take more than 30 minutes to arrive for ED consultation and may be available 8 to 23 hours a day. Nurses see a volume of patients with a ratio of approximately 1 nurse up to 10 patients. Doctors, nurses, support staff may receive continuing CME annually. There is either a lead doctor or nurse "pediatric champion."	There is a doctor dedicated to working in the ED 24/7. Doctors may see a volume of patients with a ratio of approximately 1 doctor up to 4 patients. Consultants may be physically located in the ED. Consultants are available to supervise trainee doctors or medical officers up to 24 hours a day. Doctors may come from a training background including pediatric emergency medicine. Pediatric credentialing is required. More than 3 different types of subspecialties are available including surgical or medical subspecialties. Subspecialists may take less than 30 minutes to arrive for ED consultation and are available up to 24 hours a day. Nurses see a volume of patients with a ratio of approximately 1 nurse up to 4 patients. Doctors, nurses, support staff receive CME opportunities ranging from monthly to weekly. There is both a lead doctor and nurse "pediatric champion."

Resources

Spotting the Sick Child is a resource commissioned by the United Kingdom Department of Health and Health Education England to support health professionals in the assessment of acutely sick children. Register to access free training modules and videos that teach approaches to common pediatric conditions.

1. Pang, K., Wood, P., Riordan, A., Prevatt, N., Henderson, J,, Reeves, A., Roland, D., Davies, F. (2020). Spotting the sick child [Videos]. RCPCH. https://spottingthesickchild.com/

2. IFEM. (2019). Chapter eight: The staffing of an emergency. Standards of care for children in emergency departments (pp. 45-52). https://assets.nationbuilder.com/ifem/pages/286/attachments/original/1650673696/Standards-of-Care-for-Children-in-Emergency-Departments-V3-April-2019.pdf?1650673696

3. IFEM. (2019). Chapter nine: The staffing of an emergency. Standards of Care for Children in Emergency Departments (pp. 53-60). https://assets.nationbuilder.com/ifem/pages/286/attachments/original/1650673696/Standards-of-Care-for-Children-in-Emergency-Departments-V3-April-2019.pdf?1650673696

4. RCPCH. (2018, June). Chapter 3: Workforce and training. Facing the future: Standards for children in emergency care (pp. 29-34). https://www.rcpch.ac.uk/sites/default/files/2018-06/FTFEC%20Digital%20updated%20final.pdf

5. Saidinejad, M., Shahid, S. (2020, January). Role of the Physician Pediatric Emergency Care Coordinator (PECC) in the ED. EIIC. https://emscimprovement.center/domains/pediatric-readiness-project/readiness-toolkit/readiness-toolkit-checklist/pecc/md-pecc/
